# Three-way decisions in generalized intuitionistic fuzzy environments: survey and challenges

**DOI:** 10.1007/s10462-023-10647-5

**Published:** 2024-02-06

**Authors:** Juanjuan Ding, Chao Zhang, Deyu Li, Jianming Zhan, Wentao Li, Yiyu Yao

**Affiliations:** 1 School of Computer and Information Technology, Key Laboratory of Computational Intelligence and Chinese Information Processing of Ministry of Education, Shanxi University, Taiyuan, 030006 Shanxi China; 2 School of Mathematics and Statistics, Hubei Minzu University, Enshi, 445000 Hubei China; 3 College of Artificial Intelligence, Southwest University, Chongqing, 400715 China; 4 Department of Computer Science, University of Regina, Regina, Saskatchewan S4S 0A2 Canada

**Keywords:** Granular computing, Three-way decision, Intuitionistic fuzzy set, Bibliometric analysis

## Abstract

Enhancing decision-making under risks is crucial in various fields, and three-way decision (3WD) methods have been extensively utilized and proven to be effective in numerous scenarios. However, traditional methods may not be sufficient when addressing intricate decision-making scenarios characterized by uncertain and ambiguous information. In response to this challenge, the generalized intuitionistic fuzzy set (IFS) theory extends the conventional fuzzy set theory by introducing two pivotal concepts, i.e., membership degrees and non-membership degrees. These concepts offer a more comprehensive means of portraying the relationship between elements and fuzzy concepts, thereby boosting the ability to model complex problems. The generalized IFS theory brings about heightened flexibility and precision in problem-solving, allowing for a more thorough and accurate description of intricate phenomena. Consequently, the generalized IFS theory emerges as a more refined tool for articulating fuzzy phenomena. The paper offers a thorough review of the research advancements made in 3WD methods within the context of generalized intuitionistic fuzzy (IF) environments. First, the paper summarizes fundamental aspects of 3WD methods and the IFS theory. Second, the paper discusses the latest development trends, including the application of these methods in new fields and the development of new hybrid methods. Furthermore, the paper analyzes the strengths and weaknesses of research methods employed in recent years. While these methods have yielded impressive outcomes in decision-making, there are still some limitations and challenges that need to be addressed. Finally, the paper proposes key challenges and future research directions. Overall, the paper offers a comprehensive and insightful review of the latest research progress on 3WD methods in generalized IF environments, which can provide guidance for scholars and engineers in the intelligent decision-making field with situations characterized by various uncertainties.

## Introduction

Artificial Intelligence (AI) stands as a leading-edge domain in today’s technological advancements, permeating nearly every industry and sector, with the underpinning of big data playing a pivotal role in its advancement (Buxton et al. 2008; Hanson et al. 2011; Chen et al. 2021; Wu et al. 2014). Due to the rapid and extensive fusion of information technology, the concept of big data has gained significant prominence in the Internet era. It has emerged as a highly discussed and impactful subject, offering vast opportunities to enhance individuals’ comprehension and address a wide array of challenges. Nevertheless, during the handling and examination of big data, individuals have gradually come to recognize that the issue of data volume is merely superficial. The primary concern lies in the quality and precision of the data. At this point, granular computing has become a highly valued field (Zadeh et al. 1979). Granular computing is a relatively new intelligent computing framework that emphasizes thinking styles, problem-addressing methodologies, and data processing models founded with respect to granular structures (Hobbs 1985; Zadeh 1997).

**Fig. 1:**
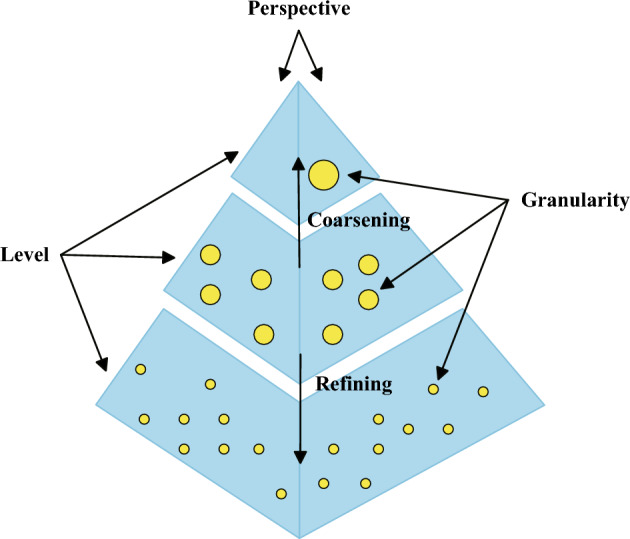
Granular computing ideas

Granular computing’s aim is to process information’s uncertainty and approximation in complex systems (Lin 1997; Xu and Li 2016). Compared to traditional computing methods, granular computing has advantages such as efficiency and flexibility, which can quickly process large-scale data and extract useful information from it (Yao et al. 2013). Granular computing (Yao 2008) is broadly categorized into two types: one is founded upon the granulation of information, such as fuzzy sets; the other is founded on multi-granularity computing, such as rough sets, 3WD, the quotient space, and cloud models. The fundamental idea of granular computing, as depicted in Fig. 1, is to break down information into finer granules from multiple levels and perspectives, or to merge information into coarser granules.

**Fig. 2:**
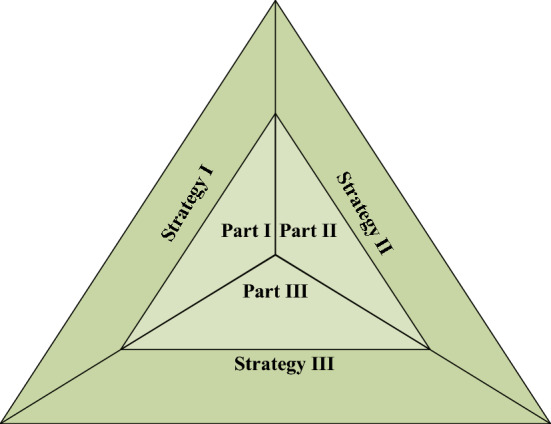
The TAO model

In recent years, Yao (2010) has made significant contributions to the field of granular computing by developing 3WD. This method proves to be an effective tool for managing ambiguous information and holds an important position among other methods in the field. Compared to two-way decisions that only consider acceptance or rejection, by incorporating a delayed decision, 3WD aligns more closely with human cognition. This method partitions the complete decision space into three distinct regions, i.e., the positive region, the negative region, and the boundary region. These regions correspond to acceptance, rejection, and delayed decisions, respectively. From both macroscopic and microscopic perspectives, 3WD can be classified into generalized and narrow decisions. Generalized 3WD primarily focuses on philosophical viewpoints. Yao (2018) initially proposed the Trisecting-Acting-Outcome (TAO) model, which divides a whole into three parts and applies different strategies to each part, which is shown in Fig. 2. Subsequently, Yao (2022) introduced the Symbols-Meaning-Value (SMV) space. In recent years, Yao (2018, 2016) has explored a series of generalized 3WD models, including interval set models, fuzzy set models, shadowed set models, and rough set models, as well as three-way clustering (Chu et al. 2020; Zhang et al. 2023b) and three-way conflict analysis (Yao 2019; Li et al. 2022). The core focus of 3WD in its narrower sense is on the decision-theoretic rough set (DTRS). DTRSs incorporate the Bayesian decision risk method into the rough set theory to identify the optimal decision solution that minimizes risk costs (Zhang et al. 2020b; Wang et al. 2021a). In summary, as a powerful instrument for managing ambiguous data, 3WD provides strong support for problem-solving in practice.

Due to the intricate nature of decision-making environments, coupled with their uncertainty and fuzziness, as well as the inherent vagueness of human cognition, the conventional 3WD theory has progressively proven insufficient to address the requirements of practical decision-making. Accordingly, it is crucial to integrate the 3WD theory with other theories to fully utilize its advantages. IFSs emerge as a formidable instrument for effectively managing data fraught with ambiguity. Its distinct advantage lies in its capacity to intricately capture and represent both uncertainty and fuzziness inherent in information, thereby offering a richer and more nuanced perspective for decision-makers. Therefore, investigations into combining the 3WD theory with the IFS theory is of great value in enhancing efficiency and accuracy of processing uncertain information and decision-making.

**Fig. 3:**
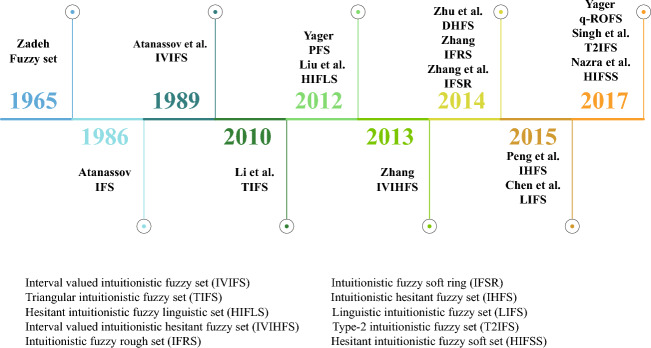
IFS and its extension

Atanassov (1986) initiated IFSs as a mathematical tool for intuitively representing fuzzy concepts in 1986, extending (Zadeh 1965) fuzzy sets by incorporating non-membership degrees and hesitant degrees. IFSs provide a more precise description of complex real-world problems while also allowing for more flexible handling of fuzzy and uncertain situations. In 2012, (Zhu et al. 2012) put forward the idea of dual hesitant fuzzy sets (DHFSs), which integrate the advantages of IFSs and hesitant fuzzy sets (HFSs). The membership degree and non-membership degree of DHFSs consist of multiple numbers, which can describe imprecise and hesitant information, with the cumulative total of membership degrees and non-membership degrees is less than or equal to 1. In 2014, (Yager 2014) introduced the form of Pythagorean fuzzy sets (PFSs) founded on IFSs, where the cumulative total of the squares of membership degrees and non-membership degrees is less than or equal to 1 with looser restrictions, providing a more flexible approach. In 2017, (Yager 2017) extended the scope of uncertainty expression beyond IFSs and PFSs by introducing q-rung orthopair fuzzy sets (q-ROFSs), which require membership degrees and non-membership degrees to satisfy a specific condition of ${\mu ^q} + {\nu ^q} \le 1$ and $q \ge 1$. IFS and its extension are shown in Fig. 3.

3WD is a potent tool for managing uncertain information, while the generalized IFS theory represents an effective mechanism for handling ambiguity, uncertainty, and complex problems. The integration of these two theories holds significant value in enhancing the efficiency and accuracy of decision-making under uncertainty. Existing literature reviews typically focus on one or the other. For instance, (Zhan et al. 2023) explored the combination of 3WD with HFSs. (Yang et al. 2023) and colleagues integrated sequential 3WD with multi-granularity learning. Meanwhile, (Liu et al. 2017) fused sentiment analysis with IFSs. (Peng and Luo 2021) conducted a bibliometric analysis of q-ROFSs. These comprehensive papers, however, primarily discuss from a single perspective, neglecting the potential benefits of synthesizing both theories. Given the aforementioned literature analysis, there is a discernible need for a scholarly article that amalgamates these two theories. Such work would offer substantial guidance for related fields, fostering the development of more robust methods for handling uncertainty and complexity.

The principal motivations with respect to this study are illustrated below:

1. 3WD theories are important methodologies that can help people deal with uncertain information to achieve more scientific and accurate decision-making.
2. IFSs are also an important mathematical tool that can help people express ambiguous concepts and ideas more clearly.
3. Existing review literature tends to focus only on the single application scenario of either the 3WD theory or IFSs, lacking in-depth exploration of their combined applications. Therefore, it becomes particularly necessary to conduct research on 3WD founded on IFSs.

The principal contributions of the paper are outlined below:

1. This paper investigates 3WD models built upon generalized IF environments and explores the value and significance of this approach in both theoretical research and practical applications.
2. The literature on the 3WD theory and IFSs is reviewed, analyzing the research trends and development directions of these two theories to provide an important reference for further studies.
3. Bibliometric analysis quantitatively examines literature using mathematical and statistical methods. In this study, the bibliometric analysis is employed to statistically analyze the research achievements of these theories in terms of theories, models, and applications. This comprehensive analysis serves as a valuable reference for scholars and practitioners in relevant disciplines.
4. The paper highlights the innovative aspects and shortcomings of existing research, pointing out that future studies should focus on a more in-depth exploration of the combination of the 3WD theory and IFSs, as well as further expanding their applications in diverse fields.

**Fig. 4:**
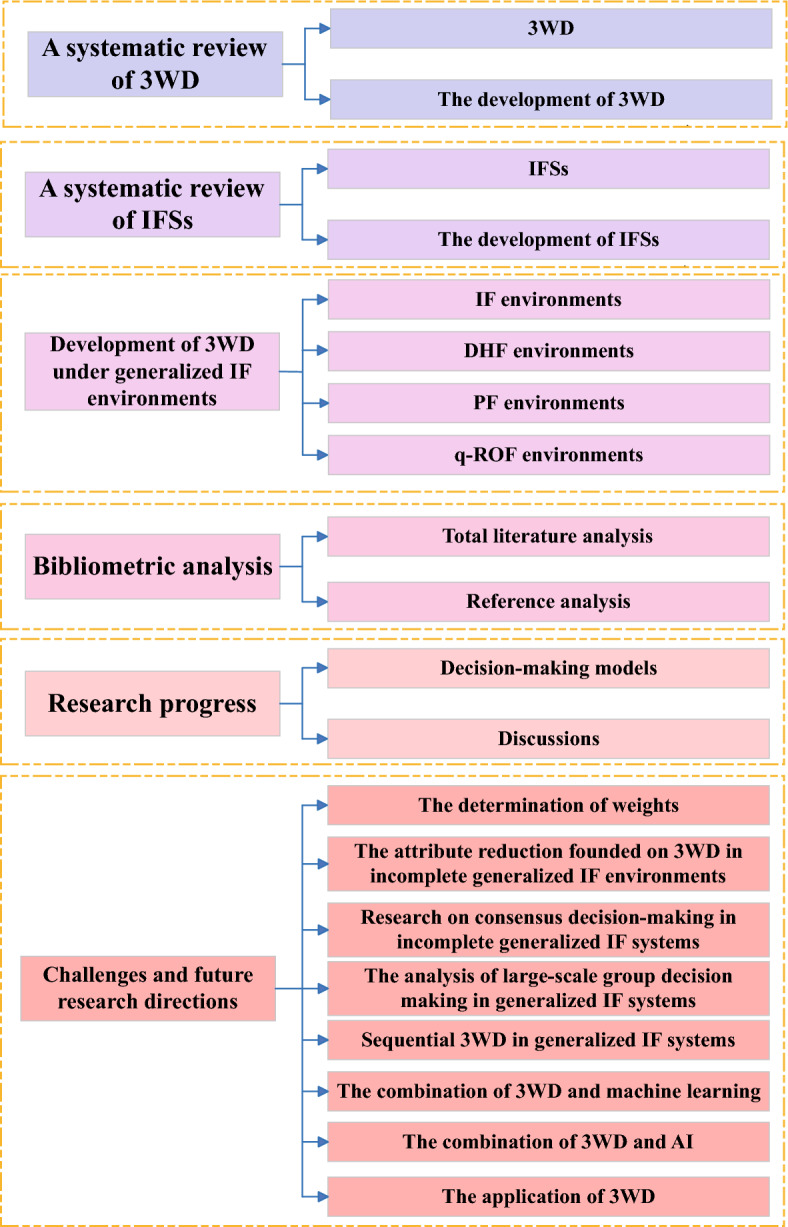
The specific framework of the paper

The goal of this paper is to explore a systematic introduction to 3WD under generalized IF environments. Therefore, the arrangement of the paper is as outlined: Sect. 2 delivers an overall picture of the theoretical framework and development process underlying 3WD; Sect. 3 presents a comprehensive introduction to the theoretical framework and iterative development process of IFSs; Sect. 4 discusses 3WD under generalized IF environments; In the next section, the bibliometric analysis of the literature on the theories is mentioned. In Sect. 6, the current research model is analyzed. Section 7 points out the future development direction. In Sect. 8, a summary of the paper is listed. Moreover, Fig. 4 illustrates the specific framework of the paper.

## A systematic review of 3WD

Moving forward, this paper will review the theory and historical development of 3WD.

### 3WD

The 3WD model, initially presented by (Yao 2010), is a decision-making approach that takes inspiration from human cognitive ability.

#### DTRSs

The 3WD method typically involves two state sets and three actions. First, the state set can be written as: $\Omega = \left\{ {V,\lnot V} \right\}$, which contains two complementary states, respectively belonging to state and not belonging to state, *V*(*P*) indicates that the object is in *V*, and $\lnot V(N)$ indicates that the object is not in *V*. The action set can be expressed as: $A = \left\{ {{a_P},{a_B},{a_N}} \right\}$. This set contains three actions for classification. ${a_P}$, ${a_B}$ and ${a_N}$ represent actions that belong to *POS*, *BND* and *NEG* respectively. The classic loss functions under two states and three actions can be listed in Table 1.

**Table 1:** Classic loss functions

	*V*(*P*)	$\lnot V(N)$
${a_P}$	${\lambda _{PP}}$	${\lambda _{PN}}$
${a_B}$	${\lambda _{BP}}$	${\lambda _{BN}}$
${a_N}$	${\lambda _{NP}}$	${\lambda _{NN}}$

In accordance with the above Table 1, ${\lambda _{*P}}$ represents the risk cost of the above three types of actions when ${x_j} \in V$; ${\lambda _{*N}}$ refers to the risk cost of the above three types of actions when ${x_j} \in \lnot V$, where $* = P,B,N$. Therefore, the expected costs under the above three types of actions can be expressed as outlined:

1 $$\begin{aligned} R({a_P}|[x]) = {\lambda _{PP}}\Pr (X|[x]) + {\lambda _{PN}}\Pr (\lnot X|[x]), \end{aligned}$$

2 $$\begin{aligned} R({a_B}|[x]) = {\lambda _{BP}}\Pr (X|[x]) + {\lambda _{BN}}\Pr (\lnot X|[x]), \end{aligned}$$

3 $$\begin{aligned} R({a_N}|[x]) = {\lambda _{NP}}\Pr (X|[x]) + {\lambda _{NN}}\Pr (\lnot X|[x]). \end{aligned}$$

Taking into account the Bayesian decision criteria, the following decision rules can be derived:

P) if $R({a_P}|[x]) \le R({a_B}|[x])$ and $R({a_P}|[x]) \le R({a_N}|[x])$, then $x \in POS(X)$,

B) if $R({a_B}|[x]) \le R({a_P}|[x])$ and $R({a_B}|[x]) \le R({a_N}|[x])$, then $x \in BND(X)$,

N) if $R({a_N}|[x]) \le R({a_P}|[x])$ and $R({a_N}|[x]) \le R({a_B}|[x])$, then $x \in NEG(X)$.

Considering a reasonable assumption that $0 \le {\lambda _{PP}} \le {\lambda _{BP}} \le {\lambda _{NP}}$ and $0 \le {\lambda _{NN}} \le {\lambda _{BN}} \le {\lambda _{PN}}$, (Yao 2010) gave the following decision rules in accordance with classic loss functions:

P1) if $\mathrm{{Pr(X|[x])}} \ge \alpha$ and $\mathrm{{Pr(X|[x])}} \ge \gamma$, then $x \in POS(X)$,

B1) if $$\beta \le \Pr (X|[x]) \le \alpha$$, then $$x \in BND(X)$$,

N1) if $\Pr (X|[x]) \le \beta$ and $\Pr (X|[x]) \le \gamma$, then $x \in NEG(X)$, where $\alpha$, $\beta$ and $\gamma$ are:

4 $$\begin{aligned} \alpha = \frac{{({\lambda _{PN}} - {\lambda _{BN}})}}{{({\lambda _{PN}} - {\lambda _{BN}}) + ({\lambda _{BP}} - {\lambda _{PP}})}}, \end{aligned}$$

5 $$\begin{aligned} \beta = \frac{{({\lambda _{BN}} - {\lambda _{NN}})}}{{({\lambda _{BN}} - {\lambda _{NN}}) + ({\lambda _{NP}} - {\lambda _{BP}})}}, \end{aligned}$$

6 $$\begin{aligned} \gamma = \frac{{({\lambda _{PN}} - {\lambda _{NN}})}}{{({\lambda _{PN}} - {\lambda _{NN}}) + ({\lambda _{NP}} - {\lambda _{PP}})}}. \end{aligned}$$

Since $0 \le \beta< \gamma < \alpha \le 1$, the above rules can be further simplified as:

P2) if $$\mathrm{{Pr(X|[x])}} \ge \alpha$$, then $$x \in POS(X)$$,

B2) if $$\beta \le \Pr (X|[x]) \le \alpha$$, then $$x \in BND(X)$$,

N2) if $$\Pr (X|[x]) \le \beta$$, then $$x \in NEG(X)$$.

The aforementioned rules signify that if an event [*x*] transpires and the conditional probability of *X* surpassing the threshold value $\alpha$ is greater, then *x* is categorized into positive domains. Assume an event [*x*] occurs, if the conditional probability of *X* falls between the threshold values $\alpha$ and $\beta$, then *x* is classified into the boundary domain. The boundary domain signifies insufficient conditions for making a decision, prompting the need for additional data collection and further evaluation. If the probability of occurrence of an event *X* is below a certain threshold value $\beta$, then *X* is classified as a negative outcome.

It is demonstrated in the above decision rules that the classic decision rules are directly applied to decision-making problems, and there are two limitations as follows:

1. The classic loss needs to be subjectively set by experts, which exerts a substantial influence on the final decision outcome.
2. Each decision matrix only corresponds to a single threshold, and it is difficult to obtain the corresponding diversity threshold for each alternative scheme. Therefore, (Jia and Liu 2019) proposed a new loss function under the fuzzy multi-criteria background, and established the decision-making-based 3WD method.

From Table 1, when ${x_j} \in V$, ${\lambda _{*P}}$ is the risk cost of taking actions; when ${x_j} \in \lnot V$, ${\lambda _{*N}}$ is the risk cost of taking actions. Suppose ${\widetilde{\lambda }_{BP}} = {\lambda _{BP}} - {\lambda _{PP}}$, ${\widetilde{\lambda }_{BP}} = {\lambda _{BP}} - {\lambda _{PP}}$, ${\widetilde{\lambda }_{PN}} = {\lambda _{PN}} - {\lambda _{NN}}$ and ${\widetilde{\lambda }_{BN}} = {\lambda _{BN}} - {\lambda _{NN}}$, the above mentioned relative loss functions can be presented in Table 2.

**Table 2:** Relative loss functions

	*V*(*P*)	$\lnot V(N)$
${a_{P}}$	0	${{{\widetilde{\lambda }}} _{PN}}$
${a_B}$	${{{\widetilde{\lambda }}} _{BP}}$	${{{\widetilde{\lambda }}} _{BN}}$
${a_N}$	${{{\widetilde{\lambda }}} _{NP}}$	0

##### Remark 1

(Yao 2010) initially developed a method for determining the threshold based on the loss function within the DTRS model. Building upon Yao’s work, (Jia and Liu 2019) extended this approach by employing relative loss functions to calculate the threshold. (Herbert and Yao 2011) proposed the game-theoretic rough set from the perspective of objective optimization, and addressed the corresponding threshold with Nash equilibrium. (Jia et al. 2014) devised an adaptive learning algorithm for threshold adjustment. (Liu et al. 2023) employed optimization techniques to determine thresholds and derive corresponding rules. (Ye and Liu 2022) approached the problem from a spatiotemporal perspective, proposing a recommendation method for threshold determination based on cost-sensitive approaches.

#### Generalized 3WD

The fundamental principle of 3WD is to separate a holistic problem into three distinct parts and employ valid strategies for each part. Guided by this principle, (Yao 2018) proposed the TAO model, which integrates effectiveness evaluation into the process of partitioning and handling, enabling quantitative assessment of the efficacy of these decisions. Furthermore, (Yao 2022) introduced the idea of the SMV space, which categorizes the overall operation into three levels: see, know, and act.

##### Definition 1

Based on the condition set, 3WD divides the set *X* into three pairwise disjointed regions via a mapping *f*, denoted by

7 $$\begin{aligned} \pi = \{ {P_1},{P_2},{P_3}\}, \end{aligned}$$

where $$f:X \rightarrow \pi$$, $$X = {P_1} \cup {P_2} \cup {P_3}$$, $${P_1} \cap {P_2} = \emptyset$$, $${P_1} \cap {P_3} = \emptyset$$ and $${P_2} \cap {P_3} = \emptyset$$.

Yao (2018, 2016) proposed a series of generalized models for 3WD. In the subsequent discussion, this paper employs interval sets, fuzzy sets, rough sets, shadowed sets, and partially ordered sets to exemplify the process of constructing a 3WD model.

1. Interval set
  8 $$\begin{aligned} {P_1}([{{\overline{I}}} ,{\underline{I}} ]) = \{ x \in X|\nu (x) \ge T\}; \end{aligned}$$
  9 $$\begin{aligned} {P_2}([{{\overline{I}}} ,{\underline{I}} ]) = \{ x \in X|F< \nu (x) < T\}; \end{aligned}$$
  10 $$\begin{aligned} {P_3}([{{\overline{I}}} ,{\underline{I}} ]) = \{ x \in X|\nu (x) \le F\}, \end{aligned}$$
  where $[{{\overline{I}}},{\underline{I}} ]$ is a subset in space, $\nu (x)$ is the evaluation function, (*T*, *F*) refers to thresholds, and $T \le F$.
2. Fuzzy set
  11 $$\begin{aligned} {P_1}({\mu _F}) = \{ x \in X|{\mu _F}(x) \ge \alpha \}; \end{aligned}$$
  12 $$\begin{aligned} {P_2}({\mu _F}) = \{ x \in X|\beta< {\mu _F}(x) < \alpha \}; \end{aligned}$$
  13 $$\begin{aligned} {P_3}({\mu _F}) = \{ x \in X|{\mu _F}(x) \le \beta \}, \end{aligned}$$
  where $(\alpha ,\beta )$ refers to thresholds, and $0 \le \beta \le \alpha \le 1$.
3. Rough set
  14 $$\begin{aligned} {P_1}(X) =\{ [x] \subseteq {{\widetilde{X}}}|x \in X\}; \end{aligned}$$
  15 $$\begin{aligned} {P_2}(X) = \{ \lnot ([x] \subseteq {{\widetilde{X}}}) \wedge \lnot ([x] \subseteq {{{{\widetilde{X}}}}^C})|x \in X\}; \end{aligned}$$
  16 $$\begin{aligned} {P_3}(X) =\{ [x] \subseteq {{{{\widetilde{X}}}}^C}|x \in X\}, \end{aligned}$$
  where $$\forall {{\widetilde{X}}} \subseteq X$$.
4. Shadowed set
  17 $$\begin{aligned} {P_1}({S_F}) = \{ x \in X|{\mu _F}(x) \ge 1 - \tau \} ; \end{aligned}$$
  18 $$\begin{aligned} {P_2}({S_F}) = \{ x \in X|\tau< {\mu _F}(x) < 1 - \tau \} ; \end{aligned}$$
  19 $$\begin{aligned} {P_3}({S_F}) = \{ x \in X|{\mu _F}(x) \le \tau \}, \end{aligned}$$
  where $$0 \le \tau \le 0.5$$.
5. Partially ordered set
  20 $$\begin{aligned} {P_1}(f) = \{ x \in X|f(x) \in {L_1}\} ; \end{aligned}$$
  21 $$\begin{aligned} {P_2}(f) = \{ x \in X|f(x) \notin {L_1} \wedge f(x) \notin {L_2}\} ; \end{aligned}$$
  22 $$\begin{aligned} {P_3}(f) = \{ x \in X|f(x) \in {L_2}\}, \end{aligned}$$
  where ${L_1}$ and ${L_2}$ are two subsets of *L*.

Additionally, generalized 3WD also includes extensions such as three-way clustering (Chu et al. 2020), three-way classification (Wu et al. 2021), and three-way spaces (Hu 2014). These approaches provide further expansion and extension of 3WD from diverse perspectives.

### The development of 3WD

As a new method for analyzing decisions under uncertain information, the 3WD model has seen significant advancements in recent years towards its theoretical expansion and practical applications. Scholars have conducted numerous studies focusing on refining the model and developing practical solutions, resulting in rich outcomes for both the theory and real-world applications.

At the level of theoretical extensions, Ciucci and Dubois (Ciucci and Dubois 2013) discussed the three-way logic problem under 3WD and the relationship among the three-way logic. (Yu et al. 2014) explored the three-way clustering problem with reference to the 3WD theory. (Hu 2014) studied the three-way space, and introduced axiomatic definitions from three aspects of decision matrices, decision conditions and decision evaluation functions. (Yao 2016) investigated the interaction between 3WD and cognitive computing. (Li et al. 2017) constructed the multigranulation three-way conceptual model via the cognitive computing framework. (Qian et al. 2020) studied multigranulation sequential 3WD. (Luo et al. 2022) took the union function and the conflict function as two evaluation functions and studied the three-way conflict model. (Sun et al. 2022) talked over the three-way clustering algorithm drew on entropy and applied it to interval prediction.

At the level of applications, (Zhu et al. 2022) proposed a 3WD approach founded upon regret theory. (Lu et al. 2022) applied 3WD to the watershed ecological compensation model and analyzed the government’s decisions. (Shen et al. 2022) integrated 3WD with the secure semi-supervised support vector machine. (Zhang et al. 2018) explored a new multi-label classification model and adopted 3WD for ensemble learning. (Shen et al. 2020) studied hierarchical classification model based on 3WD. (Qian et al. 2022) integrated the sequential 3WD approach with the multigranulation model and applied it to hierarchical classification. (Xu et al. 2023) discussed flow calculations under incomplete mixed information systems. (Li et al. 2021) used the 3WD method to make decision judgment of epidemic resumption against the backdrop of fuzzy linguistic double hierarchy. (Han et al. 2022) considered 3WD based upon the probabilistic linguistic setting and used it to deal with air quality assessments.

At the level of model improvement, (Zhang et al. 2020b) analyzed the adjustable hesitant fuzzy linguistic DTRS model, in which decision-makers can express decision risks of information fusion via risk coefficients. (Wang et al. 2020b) explored a 3WD method based upon prospect theory, which describes experts’ risk attitude under uncertain environment through prospect theory. (Zhang and Yao 2017) studied the 3WD model founded upon Gini coefficient, which divides the three-way areas. (Wang et al. 2022c) put forward a series of 3WD approaches built upon regret theory under the HF environment. (Yang et al. 2020) introduced the Gaussian function into 3WD and studied a multi-level domain sequential model. (Huang et al. 2020b) combined 3WD with incremental algorithm to discuss the three-way neighborhood model based upon dynamic incomplete mixed data. (Zhang et al. 2019) integrated 3WD with convolutional neural networks for emotion classification and proved its effectiveness. (Shen et al. 2022) used support vector machine to make rejection inference judgment, and further used 3WD filtering to filter the excluded samples.

The 3WD model has been widely adopted as a tool for addressing complex group decision-making problems, leading to significant research by numerous academics. For instance, (Lei et al. 2020) designed the multigranulation behavioral DTRS model with two universes to tackle decision-making issues. (Liang et al. 2015) examined multi-attribute group decision-making (MAGDM) founded on DTRSs within the context of linguistic assessments. (Wang et al. 2021b) expanded the application of group relations to social networks and investigated the external influences of social trust networks. (Luo et al. 2021) put forward a multigranulation decision-making approach founded upon inclusion measures with hesitant fuzzy linguistic values. (Sun et al. 2018) explored probabilistic rough sets and DTRSs founded on linguistic terms and then analyzed MAGDM problems founded on linguistic terms. (Hu et al. 2017) discussed linguistic DTRSs founded on the cloud model. (Wang et al. 2020a) discussed a sequential three-way MAGDM approach founded upon expert social influence to achieve consensus.

To sum up, 3WD has made great progress in theory, model improvement and application.

## A systematic review of IFSs

In what follows, this paper will review the theory and historical development of IFSs.

### IFSs

IFSs are a type of reasoning method built upon the fuzzy set theory and an enhanced edition of conventional fuzzy sets. Compared to conventional fuzzy sets, IFSs can handle more complex, fuzzy, and uncertain information.

#### Definition 2

(Atanassov 1986) Assume *X* is the universe, then an IFS can be described as:

23 $$\begin{aligned} A = \{ < x,{\mu _a}(x),{\nu _a}(x) > |x \in U\}, \end{aligned}$$

where $\mu$ and $\nu$ indicate the membership degree and the non-membership degree, and meet the requirements of $0 \le {\mu _a}(x) \le 1$, $0 \le {\nu _a}(x) \le 1$ and $0 \le {\mu _a}(x) + {\nu _a}(x) \le 1$. Additionally, ${\pi _a}(x) = 1 - {\mu _a}(x) - {\nu _a}(x)$ is the hesitant degree, and $A(x) = ({\mu _a}(x),{\nu _a}(x))$ is labeled as an IF number.

Subsequently, this paper lists common operating guidelines for IFSs.

#### Definition 3

(Atanassov 1986; Xu 2007a) The operational rules of any two IF numbers ${a_1}$ and ${a_2}$ are:

1. $${a_1} \oplus {a_2} = {{\mu _1} + {\mu _2} - {\mu _1}{\mu _2},{\nu _1}{\nu _2} }$$;
2. $$\lambda {a_1} = (1 - {(1 - {\mu _1} )^\lambda },{{\nu _1} ^\lambda })$$;
3. $${{a_1}^\lambda } = ({{\mu _1} ^\lambda },1 - {(1 - {\nu _1})^\lambda })$$;
4. $${{a_1}^c} = ({\nu _1},{\mu _1})$$;
5. $${a_1} \otimes {a_2} = { {\mu _1}{\mu _2},{\nu _1} + {\nu _2} - {\nu _1}{\nu _2} }$$;
6. $${a_1} \oslash {a_2} = \{ \min (1,\frac{{{\mu _1}}}{{{\mu _2}}}),\max (0,\frac{{{\nu _1} - {\nu _2}}}{{1 - {\nu _2}}})\}$$.

Chen and Tan (1994) and Hong and Choi (2000) recommended definitions for the score function and the accuracy function, respectively, with the aim of facilitating the assessment of the magnitude of IF numbers.

#### Definition 4

(Chen and Tan 1994; Hong and Choi 2000) For an IF number *a*, the IFS score function and exact function are denoted by

24 $$\begin{aligned} s(a) = \mu - \nu ; \end{aligned}$$

25 $$\begin{aligned} h(a) = \mu + \nu . \end{aligned}$$

### The development of IFSs

The IFS framework is renowned for its prowess in handling ambiguous data. One of its key advantages is the ability to provide a broader spectrum of possible choices, thereby enhancing the decision-making process. By expanding flexibility, IFSs empower decision-makers to navigate uncertain scenarios with greater precision and consider a wider range of potential solutions. Consequently, the IFS theory is a valuable tool for addressing complex problems that demand nuanced handling of imprecise information. Ultimately, it leads to more informed and effective decision-making by enabling a more comprehensive representation of uncertainty and fuzziness within data.

IFS, pioneered by (Atanassov 1986), serves as an extension of the classic fuzzy set theory. It also considers membership, non-membership and hesitant degrees, and can allow for a more pliable method of conveying uncertainty information. In the last few years, IFSs have made great evolve in both theory and practice.

First, the paper introduces the research of IFSs in its own theory. Chen and Tan (1994) studied the definition of IF score function, and compared the size of IF numbers with the function. Hong and Choi (2000) discussed the exact IF function, so as to further compare the size of IF numbers based on the scoring function. Burillo and Bustince (1996) first brought the notion of entropy into the IFS and proposed the concept of IF entropy. Xu (2007a) studied IF weighted averaging operators. Xu (2007b) first proposed a series of IF preference relation concepts. Xu and Yager (2006) then explored IF weighted geometric operators. Li and Cheng (2002) and Hung and Yang (2004) explored the similarity measure of IFSs.

Then this paper will introduce the extension theory built upon IFSs.

Interval-valued intuitionistic fuzzy sets (IVIFSs) are a mathematical approach for processing uncertainty and fuzziness. Based on the interval theory, it represents each variable as an interval number rather than a point value, making the expression of uncertain information more reliable.

#### Definition 5

(Atanassov and Gargov 1989) Assume *X* is the universe, and an IVIFS can be described as:

26 $$\begin{aligned} {{\widetilde{A}}} = \left\{ {\left\langle {x,[\overline{{\mu _{{{\widetilde{a}}}}}} \left( x \right) ,\underline{{\mu _{\widetilde{a}}}} \left( x \right) ],[\overline{{\nu _{{{\widetilde{a}}}}}} \left( x \right) ,\underline{{\nu _{{{\widetilde{a}}}}}} \left( x \right) ]} \right\rangle \left| {x \in X} \right. } \right\} , \end{aligned}$$

where $[\overline{{\mu _{{{\widetilde{a}}}}}} \left( x \right) ,\underline{{\mu _{{{\widetilde{a}}}}}} \left( x \right) ]$ and $[\overline{{\nu _{{{\widetilde{a}}}}}} \left( x \right) ,\underline{{\nu _{{{\widetilde{a}}}}}} \left( x \right) ]$ represent the membership interval valued degree and non-membership interval valued degree, and $\left\langle {[\overline{{\mu _{{{\widetilde{a}}}}}} \left( x \right) ,\underline{{\mu _{{{\widetilde{a}}}}}} \left( x \right) ],[\overline{{\nu _{{{\widetilde{a}}}}}} \left( x \right) ,\underline{{\nu _{{{\widetilde{a}}}}}} \left( x \right) ]} \right\rangle$ is titled an IVIF number. In an IVIFS, for each $x \in X$, $\overline{{\mu _{{{\widetilde{a}}}}}} \left( x \right) + \overline{{\nu _{{{\widetilde{a}}}}}} \left( x \right) \in [0,1]$, $\overline{{\mu _{{{\widetilde{a}}}}}} \left( x \right) \in [0,1]$, $\overline{{\nu _{{{\widetilde{a}}}}}} \left( x \right) \in [0,1]$. Additionally, the paper simplifies $\left\langle {[\overline{{\mu _{{{\widetilde{a}}}}}} \left( x \right) ,\underline{{\mu _{\widetilde{a}}}} \left( x \right) ],[\overline{{\nu _{{{\widetilde{a}}}}}} \left( x \right) ,\underline{{\nu _{{{\widetilde{a}}}}}} \left( x \right) ]} \right\rangle$ to $\left\langle {[{{\overline{\mu }}},{{\underline{\mu }}} ],[{{\overline{\nu }}},{{\underline{\nu }}} ]} \right\rangle$.

#### Definition 6

(Atanassov and Gargov 1989) Given two IVIF numbers ${{{\widetilde{a}}}_1}$ and ${{{\widetilde{a}}}_2}$, where $\lambda$ is a positive real number, their calculation rules are as specified below:

1. $${{\widetilde{a}}}_1^c = ([\overline{{\nu _1}},\underline{{\nu _1}} ],[\overline{{\mu _1}},\underline{{\mu _1}} ])$$;
2. $${{{\widetilde{a}}}_1}^\lambda = ([{\overline{{\mu _1}} ^\lambda },{\underline{{\mu _1}} ^\lambda }],[1 - {(1 - \overline{{\nu _1}} )^\lambda },1 - {(1 - \underline{{\nu _1}} )^\lambda }])$$;
3. $${{{\widetilde{a}}}_1} \oplus {{{\widetilde{a}}}_2} = ([\overline{{\mu _1}} + \overline{{\mu _2}} - \overline{{\mu _1}} \overline{{\mu _2}},\underline{{\mu _1}} + \underline{{\mu _2}} - \underline{{\mu _1}} \underline{{\mu _2}} ],[\overline{{\nu _1}} \overline{{\nu _2}},\underline{{\nu _1}} \underline{{\nu _2}} ])$$;
4. $$\lambda {{{\widetilde{a}}}_1} = ([1 - {(1 - \overline{{\mu _1}} )^\lambda },1 - {(1 - \underline{{\mu _1}} )^\lambda }],[{\overline{{\nu _1}} ^\lambda },{\underline{{\nu _1}} ^\lambda }])$$;
5. $${{{\widetilde{a}}}_1} \otimes {{{\widetilde{a}}}_2} = ([\overline{{\mu _1}} \overline{{\mu _2}},\underline{{\mu _1}} \underline{{\mu _2}} ],[\overline{{\nu _1}} + \overline{{\nu _2}} - \overline{{\nu _1}} \overline{{\nu _2}},\underline{{\nu _1}} + \underline{{\nu _2}} - \underline{{\nu _1}} \underline{{\nu _2}} ])$$.

Linguistic intuitionistic fuzzy sets (LIFSs) represent information in a qualitative form, using linguistic variables to express membership degrees and non-membership degrees. Compared with the quantitative expression of precise numbers, LIFSs are more flexible and can demonstrate decision-makers’ preferences and oppositions to objective things. The advantage of LIFSs is that they have a certain degree of fuzziness, which can be better applied to complex decision analysis. By using LIFSs, decision-making information can be expressed more accurately in terms of its meaning.

#### Definition 7

(Chen et al. 2015) Assume ${s_\mu },{s_\nu } \in S = \{ {s_i}|i = 0,1, \cdots ,2t\}$, and an LIFS can be described as:

27 $$\begin{aligned} {{\widehat{A}}} = \{ < x,{s_\mu },{s_\nu } > |x \in X\}, \end{aligned}$$

where ${s_\mu }$ and ${s_\nu }$ represent the linguistic membership degree and linguistic non-membership degree, and it satisfies that ${s_0} \le {s_\mu } \oplus {s_\nu } \le {s_{2t}}$. $< {s_\mu }(x),{s_\nu }(x)>$ is called IFL number. For convenience, the paper simplifies $< {s_\mu }(x),{s_\nu }(x) >$ to $({s_\mu },{s_\nu })$.

#### Definition 8

(Chen et al. 2015) Given two LIF numbers ${{{\widehat{a}}}_1}$ and ${{{\widehat{a}}}_2}$, where $\lambda > 0$, the calculation of these values follows these rules:

1. $${{\widehat{a}}}_1^c = ({s_{{\nu _1}}},{s_{{\mu _1}}})$$;
2. $${{{\widehat{a}}}_1}^\lambda = (f{({s_{{\mu _1}}})^\lambda },1 - (1 - f{({s_{{\nu _1}}})^\lambda })$$;
3. $$\lambda {{{\widehat{a}}}_1} = (1 - {(1 - f({s_{{\mu _1}}}))^\lambda },f{({s_{{\nu _1}}})^\lambda })$$;
4. $${{{\widehat{a}}}_1} \oplus {{{\widehat{a}}}_2} = (1 - (1 - f({s_{{\mu _1}}}))(1 - f({s_{{\mu _2}}})),f({s_{{\nu _1}}})f({s_{{\nu _2}}}))$$;
5. $${{{\widehat{a}}}_1} \otimes {{{\widehat{a}}}_2} = (f({s_{{\mu _1}}})f({s_{{\mu _2}}}),f({s_{{\nu _1}}}) + f({s_{{\nu _2}}}) - f({s_{{\nu _1}}})f({s_{\nu 2}})$$.

where ${s_\mu } = f({s_\mu }) = \frac{\mu }{{2t}}$ and ${s_\nu } = f({s_\nu }) = \frac{\nu }{{2t}}$, and $f({s_i}) = \frac{i}{{2t}}(i = 0,1, \cdots ,2t)$ is the linguistic scale function.

DHFSs are a generalized form of IFSs and HFSs. In DHFSs, membership degrees and non-membership degrees are represented by some possible values between 0 and 1, which can offer a more accurate and comprehensive description of a decision-maker’s evaluative stance.

#### Definition 9

(Zhu et al. 2012) Assume *X* is the universe, and a DHFS can be described as:

28 $$\begin{aligned} D = \left\{ {\left\langle {x,{\mu _D}\left( x \right) ,{\nu _D}\left( x \right) } \right\rangle \left| {x \in X} \right. } \right\} , \end{aligned}$$

where ${\mu _{{{\mathcal {D}}}}}(x)$ and ${\nu _{{{\mathcal {D}}}}}(x)$ are sets of several possible values in [0, 1], representing the membership degree and the non-membership degree, and $d(x) = ({\mu _\mathcal{D}}(x),{\nu _{{{\mathcal {D}}}}}(x))$ is titled a DHF element. For each $x \in U$, let $\alpha \in {\mu _{{{\mathcal {D}}}}}(x)$, $\beta \in {\nu _\mathcal{D}}(x)$, ${\alpha ^ + } = \max \{ \alpha |\alpha \in {\mu _\mathcal{D}}(x)\}$ and ${\beta ^ + } = \max \{ \beta |\beta \in {\nu _\mathcal{D}}(x)\}$, then $0 \le \alpha$, $\beta \le 1$ and $0 \le {\alpha ^ + } + {\beta ^ + } \le 1$ are satisfied. For convenience, this paper simplifies $({\mu _D}(x),{\nu _D}(x))$ to $(\mu ,\nu )$.

In an effort to facilitate the operation of different DHFSs, (Zhang et al. 2017) proposed the following two stipulations: First, it is stipulated that the elements of $\mu$ and $\nu$ in the DHF element *d* are arranged in an ascending order respectively. It is assumed that the $i-th$ element in $\mu$ is represented as ${\mu ^{\tau (i)}}$, and the $i-th$ element in $\nu$ is represented as ${\nu ^{\tau (i)}}$. Second, it is specified that for two DHF elements ${d_1}$ and ${d_2}$, let ${l_\mu }$ be the number of elements of $\mu$, ${l_\mu } = \max \{ {l_{{\mu _1}}},{l_{{\mu _2}}}\}$, and ${l_\nu }$ be the number of elements of $\nu$. For two DHF elements ${d_1}$ and ${d_2}$, if ${l_{{\mu _1}}} \ne {l_{{\mu _2}}}$ or ${l_{{\nu _1}}} \ne {l_{{\nu _2}}}$, the minimum value is incorporated into the DHF element with a limited number of elements for extensions until the number of elements in both DHF membership and non-membership degrees is equal.

#### Definition 10

(Zhu et al. 2012) Given two DHF elements ${d_1}$ and ${d_2}$, where $\lambda > 0$, their calculation rules are as outlined below:

1. $$d_1^c = \{ \nu _1^{\tau (i)},\mu _1^{\tau (i)}\}$$;
2. $${d_1}^\lambda = \{ {(\mu _1^{\tau (i)})^\lambda },1 - {(1 - \nu _1^{\tau (i)})^\lambda }\}$$;
3. $$\lambda {d_1} = \{ 1 - {(1 - \mu _1^{\tau (i)})^\lambda },{(\nu _1^{\tau (i)})^\lambda }\}$$;
4. $${d_1} \oplus {d_2} = \{ \{ \mu _1^{\tau (i)} + \mu _2^{\tau (i)} - \mu _1^{\tau (i)}\mu _2^{\tau (i)}\},\{ \nu _1^{\tau (i)}\nu _2^{\tau (i)}\} \}$$;
5. $${d_1} \otimes {d_2} = \{ \{ \mu _1^{\tau (i)}\mu _2^{\tau (i)}\},\{ \nu _1^{\tau (i)} + \nu _2^{\tau (i)} - \nu _1^{\tau (i)}\nu _2^{\tau (i)}\} \}$$;
6. $${d_1} \oslash {d_2} = \{ \max \{ 1,\frac{{\mu _1^{\tau i)}}}{{\mu _2^{\tau (i)}}}\},\min \{ 0,\frac{{\nu _1^{\tau (i)} - \nu _2^{\tau (i)}}}{{1 - \nu _2^{\tau (i)}}}\} \}$$.

PFSs are a generalized form of IFSs, which have advantages in more precisely characterizing uncertainty in the objective world.

#### Definition 11

(Yager 2014) Assume *X* is the universe, and a PFS can be described as:

29 $$\begin{aligned} P = \left\{ {\left\langle {x,{\mu _P}\left( x \right) ,{\nu _P}\left( x \right) } \right\rangle \left| {x \in X} \right. } \right\} , \end{aligned}$$

where ${\mu _P}(x):X \rightarrow [0,1]$ and ${\nu _P}(x):X \rightarrow [0,1]$ represent membership degrees and non-membership degrees respectively. For any $x \in X$, $0 \le {({\mu _P}(x))^2} + {({\nu _P}(x))^2} \le 1$. ${\pi _P}(x) = \sqrt{1 - {{({\mu _P}(x))}^2} - {{({\nu _P}(x))}^2}}$ symbolizes hesitant degree, and $p = ({\mu _P}(x),{\nu _P}(x))$ is called a PF number. For convenience, this paper simplifies $({\mu _P}(x),{\nu _P}(x))$ to $(\mu ,\nu )$.

In what follows, this paper presents a list of operational rules for PFSs.

#### Definition 12

(Yager 2014; Peng and Selvachandran 2019; Zhang et al. 2018) Given two PF numbers ${p_1}$ and ${p_2}$, where $\lambda$ is a positive real number, their calculation rules are as outlined below:

1. $$p_1^c = ({\nu _1},{\mu _1})$$;
2. $${p_1}^\lambda = ({\mu _1}^\lambda ,\sqrt{1 - {{(1 - {\nu _1}^2)}^\lambda }} )$$;
3. $$\lambda {p_1} = (\sqrt{1 - {{(1 - {\mu _1}^2)}^\lambda }},{\nu _1}^\lambda )$$;
4. $${p_1} \oplus {p_2} = (\sqrt{{\mu _1}^2 + {\mu _2}^2 - {\mu _1}^2{\mu _2}^2},{\nu _1}{\nu _2})$$;
5. $${p_1} \otimes {p_2} = ({\mu _1}{\mu _2},\sqrt{{\nu _1}^2 + {\nu _2}^2 - {\nu _1}^2{\nu _2}^2} )$$;
6. $${p_1} \oslash {p_2} = (\min (0,\frac{{{\mu _1}}}{{{\mu _2}}}),\max (0,\sqrt{\frac{{{\nu _1}^2 - {\nu _2}^2}}{{1 - {\nu _2}^2}}} ))$$.

q-ROFSs are a generalized fuzzy set that have the aptitude to tackle uncertainty and fuzziness in problems, whereas also being able to represent uncertain information in real world more effectively. The q-ROFS theory provides a flexible approach for handling various types of uncertain information, including randomness, fuzziness, and others. By adjusting its parameters, q-ROFSs are capable of satisfying different demands in various scenarios, thus providing a more precise representation.

#### Definition 13

(Yager 2017) Assume *X* is the universe, and a q-ROFS can be described as:

30 $$\begin{aligned} B = \{ < x,{\mu _b}(x),{\nu _b}(x) > |x \in X\}, \end{aligned}$$

where ${\mu _b}(x):X \rightarrow [0,1]$ and ${\nu _b}(x):X \rightarrow [0,1]$ represent membership degree and non-membership degree respectively. For any $x \in X$, ${({\mu _b}(x))^q} + {({\nu _b}(x))^q} \le 1$ and $q \ge 1$. $b = ({\mu _b}(x),{\nu _b}(x))$ is called a q-ROF number. For convenience, this paper simplifies $({\mu _b}(x),{\nu _b}(x))$ to $(\mu ,\nu )$.

Additionally, the operational rules for q-ROFSs are listed below.

#### Definition 14

(Yager 2017; Zhang et al. 2021a) Given two q-ROF numbers ${b_1}$ and ${b_2}$, where $\lambda > 0$, their calculation rules are as specified below:

1. $$b_1^c = ({\nu _1},{\mu _1})$$;
2. $${b_1}^\lambda = ({\mu _1}^\lambda ,\root q \of {{1 - {{(1 - {\nu _1}^q)}^\lambda }}})$$;
3. $${b_1} \oplus {b_2} = (\root q \of {{{\mu _1}^q + {\mu _2}^q - {\mu _1}^q{\mu _2}^q}},{\nu _1}{\nu _2})$$;
4. $${b_1} \otimes {b_2} = ({\mu _1}{\mu _2},\root q \of {{{\nu _1}^q + {\nu _2}^q - {\nu _1}^q{\nu _2}^q}})$$;
5. $$\lambda {b_1} = (\root q \of {{1 - {{(1 - {\mu _1}^q)}^\lambda }}},{\nu _1}^\lambda )$$;
6. $${b_1} \oslash {b_2} = (\min (1,\frac{{{\mu _1}}}{{{\mu _2}}}),\max (0,\root q \of {{\frac{{{\nu _1}^q - {\nu _2}^q}}{{1 - {\nu _2}^q}}}}))$$.

In addition to the above theories, the extended theory of IFSs also includes: T2IFSs (Singh and Garg 2017), IFSRs (Zhang 2012b), TIFSs (Li et al. 2010), IHFSs (Peng et al. 2015), IVIHFSs (Zhang 2013), HIFLSs (Liu et al. 2014), HIFSSs (Nazra et al. 2017), IFRSs (Zhang 2012a).

Finally, the application of IFSs is introduced. Yang et al. (2021) studied two bilinear programming models and applied them to address ecological management issues. Liang et al. (2022a) put forward a time-varying IF model based on case-based reasoning and employed it to tackle forest fires. Wang et al.(2023c) studied an interpretable IF reasoning model and applied it to stock prediction. Bai et al. (2023) investigated decision-making methods founded upon incomplete IF behavior and applied it to water quality inspection. Ghaderi et al. (2020) introduced a decision model rooted in the DEA methodology and applied it to a real-world case study involving a manufacturing company. Mousavi et al. (2015) utilized the IF logic to facilitate the selection of suitable projects. Gitinavard and Akbarpour Shirazi (2018) validated the efficacy of the IF modified group complex proportional method by applying it to the equipment selection challenge within manufacturing enterprises. Hussain et al. (2023) employed the IF Aczel-Alsina Heronian mean operators for the evaluation of solar cells. Hussain et al. (2022a) utilized the IF Hamy mean operators in the assessment of tourism industry development.

## 3WD under generalized IF environments

Both IFSs and 3WD can describe the uncertainty in decision-making problems, thus many scholars have combined them to do meaningful research.

### IF environments

Gao et al. (2020) recommended a target risk evaluation approach founded upon 3WD within IF environments, which calculated conditional probability mainly through the TOPSIS method. Pang et al. (2020) studied a data-driven approach in the idea of interval-valued linguistic uncertainties. Liu et al. (2021) explored the aggregate collective loss function based on group consensus under IF environments. Jiang and Hu (2021) discussed three investment decisions under the environment of IF MAGDM. Wang et al. (2022a) investigated the 3WD model constructed built upon probabilistic dominance relations under IF environments. Song et al. (2022) used the VIKOR method for grey correlation analysis to confirm the conditional probability in a mixed MAGDM problem. Dai et al (2023) created a new IF loss function based on IF concepts.

### DHF environments

Liang et al. (2017) designed a DTRS model for DHF environments, and subsequently designed two methods for deriving 3WD based on the new model. The first method is a general approach that utilizes score functions and accuracy functions of DHF elements. The second method involves a probability metric sorting approach using random strategies. Liang et al. (2020) explored the risk preference of DHF 3WD models, and calculated the conditional probability founded on DHF entropy and cross entropy measures. Feng et al. (2023) suggested the idea of the DHF case table and utilized the CRITIC method to count attribute weights. Additionally, agents were classified based on the Bayesian minimum risk theory. Subsequently, the correlation between problems was leveraged to identify conflict characteristics and divide them into three groups. Zhang et al. (2023a) discussed the Parkinson’s disease using an incomplete 3WD model.

### PF environments

Liang et al. (2018) constructed a novel approach by utilizing PF numbers to offer an innovative explanation of the loss function. They subsequently delved into the PF DTRS model, followed by the implementation of the TOPSIS method to determine conditional probabilities. Finally, they demonstrated the efficacy of their methodology via a case study involving research and development projects. Zhang et al. (2022) analyzed the adjustable multigranulation probabilistic rough sets within the incomplete PF environment. Du et al. (2022) performed a study on conflict distance and conflict function using PF numbers. They subsequently developed a DTRS model for PF contexts founded upon the conflict function. The efficacy of the model was then demonstrated via the analysis of local government governance cases. Zhang et al. (2021b) examined the loss function using three descriptions and defined the expected loss function. Additionally, they developed four decision rules for the expected loss function and investigated a group decision-making model.

### q-ROF environments

Liang and Cao (2019) utilized the project-based distance metric and TOPSIS approach to make group decision-making, and subsequently examined group decisions established on the q-ROF DTRS. Zhang et al. (2021a) developed a family of multigranulation q-ROF probabilistic rough set models.

## Bibliometric analysis

Bibliometric analysis (Peng and Dai 2018; Peng and Luo 2021) is a quantitative method used to evaluate and analyze scientific literature. It involves the use of statistical and mathematical techniques to assess various aspects of scholarly publications, such as the amount of publications, citations, authors, journals, and keywords. Bibliometric analysis (Zyoud and Fuchs-Hanusch 2017; Fernandes et al. 2023) is a valuable tool for identifying trends, patterns, and relationships within a research field. It facilitates the evaluation of the impact of individual publications or researchers.

### Total literature analysis

To facilitate a more intuitive understanding of the progression of 3WD and 3WD in generalized IF environments, this paper made a statistical analysis of relevant literature from six aspects:

1. Publication and citation frequency
2. Research areas
3. Highly cited papers
4. Co-authorship network
5. Co-occurrence overlay
6. Citation

Web of Science^1^ is an essential resource for anyone involved in scientific research or academic publishing. With its vast collection of high-quality scientific literature and powerful search and analysis tools, it provides a valuable platform for discovery and collaboration in the scientific community. The core of the Web of Science includes SCI, SSCI and AHCI, three index libraries covering the literature in the three major domains of natural sciences, social sciences and humanities. The influence factor of its introduction is the key standard of paper quality. Consequently, this paper utilizes Web of Science database for literature statistical analysis.

#### Publication and citation frequency

**Fig. 5:**
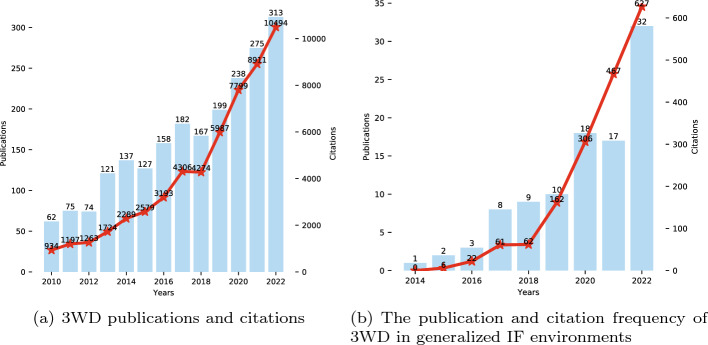
The publications and citations

Using the keywords “three-way decision”, “decision-theoretic rough set” and “probabilistic rough set”, an inquiry was performed on the Web of Science primary repository on September 10, 2023, resulting in 2723 records with a high citation frequency of 65,524. These keywords were then combined with “intuitionistic fuzzy”, “dual hesitant fuzzy”, “pythagorean fuzzy”, and “q-rung orthopair fuzzy” for further searches. Figure 5 shows the publication and citation frequency of 3WD, with (a) displaying the publication and citation frequency of 3WD between 2010-2022 and (b) displaying the publication and citation frequency of 3WD in generalized IF environments between 2014-2022.

From the gradual upward trend in Fig. 5, it can be observed that both of these themes have attracted much attention in recent years.

#### Research areas

**Fig. 6:**
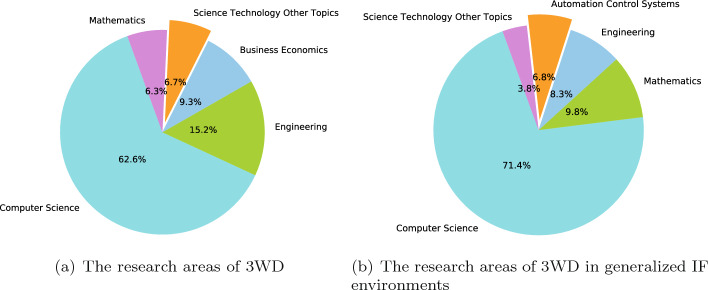
The research areas

Next, statistical analysis was conducted on the main research areas of 3WD and 3WD in generalized IF environments. As is illustrated in the Fig. 6.

Referring to Fig. 6, it is evident that computer science is the main research direction in these two fields.

#### Highly cited papers

After screening the database, 29 highly cited papers were identified. Table 3 lists authors with more than two highly cited papers, with two highly cited papers founded on the generalized IF environments, namely references (Liang et al. 2018), (Lang et al. 2020) and (Liu et al. 2022).

**Table 3:** Authors of highly cited papers

Author	Number of records
Yao Yiyu	7
Zhan Jianming	5
Huang Bing	4
Qian Yuhua	3
Li Huaxiong	3

According to Table 3, among the authors of high-level papers, Yao Yiyu ranks the first, followed by Zhan Jianming in the second place, respectively.

#### Co-authorship networks

**Fig. 7:**
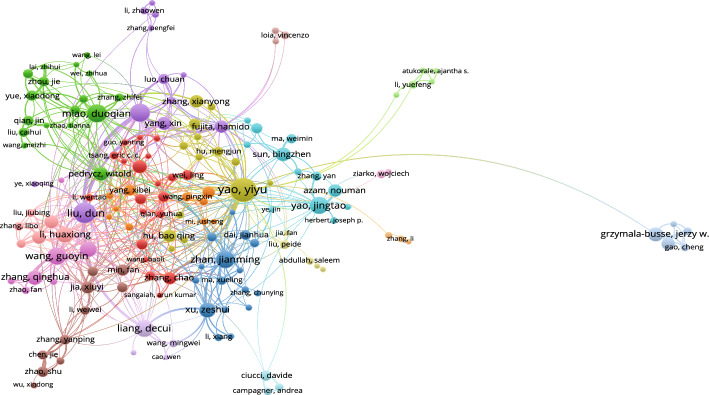
Co-authorship networks for 3WD

**Fig. 8:**
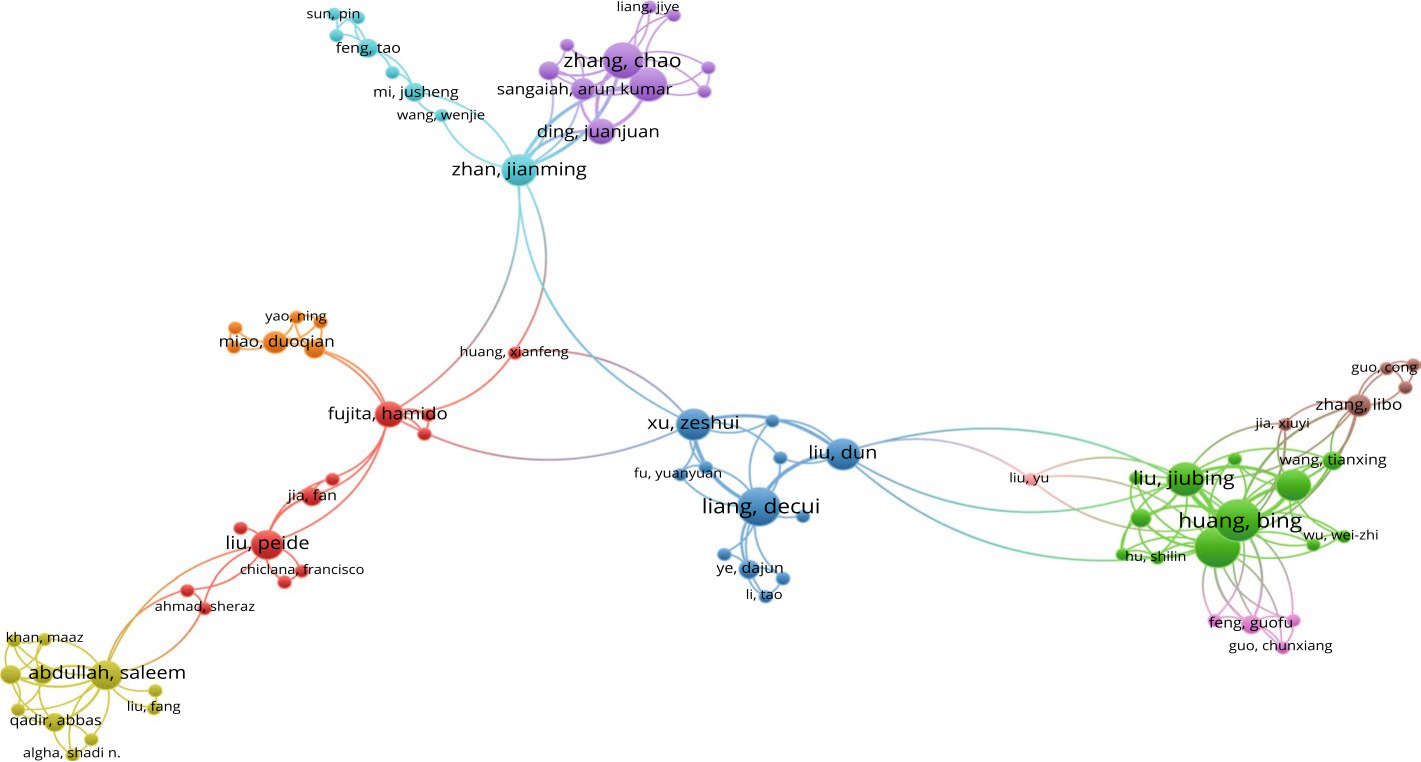
Co-authorship networks for 3WD in generalized IF environments

Founded on the data as of September 10, 2023, the co-authorship network diagram based on 3WD in Fig. 7 and the co-authorship network diagram based on 3WD in generalized IF environments in Fig. 8 can be obtained.

According to Fig. 7, the collaboration among Yao Yiyu, Liu Dun, Miao Duoqian, Li Tianrui, Yao Jingtao, and Zhan Jianming is particularly close in 3WD domains.

Referring to Fig. 8, the collaboration among Liang Decui, Liu Dun, Xu Zeshui, Li Huaxiong and Huang Bing is particularly close in 3WD in generalized IF environment domains.

#### Co-occurrence overlay

**Fig. 9:**
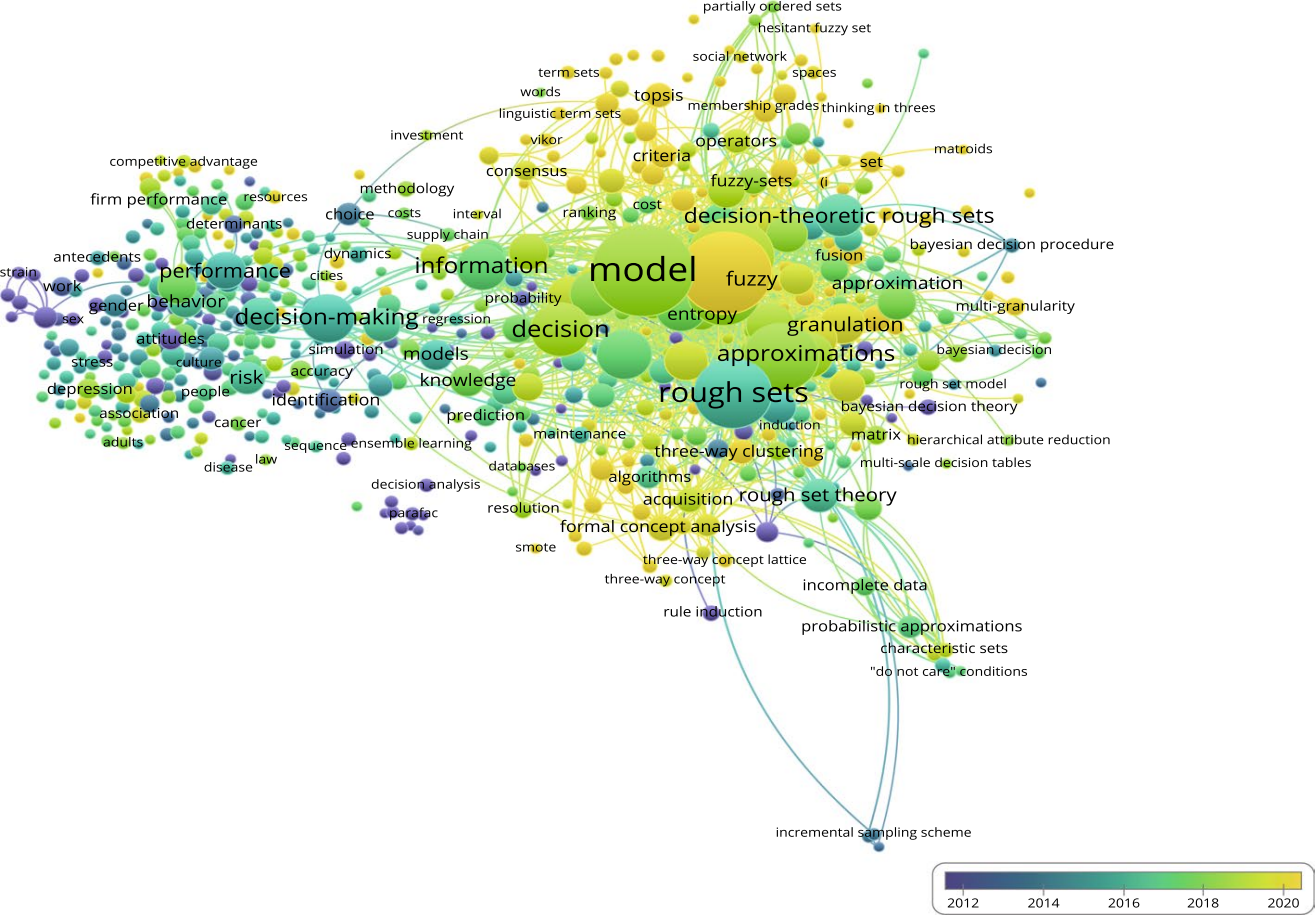
Co-occurrence overlay for 3WD

**Fig. 10:**
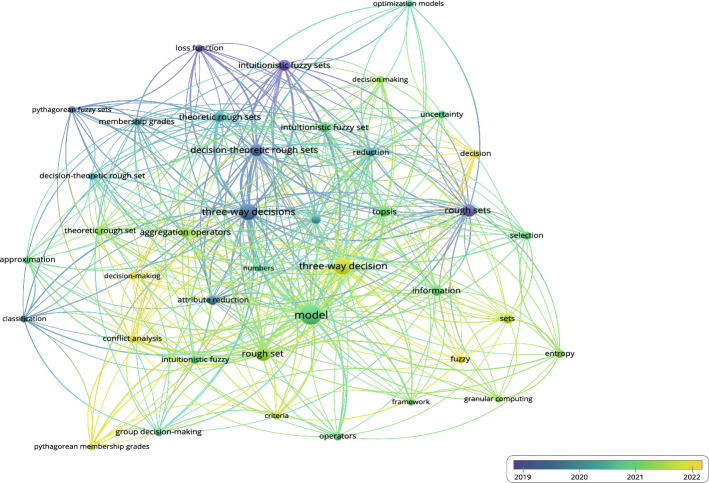
Co-occurrence overlay for 3WD in generalized IF environments

Next, the co-occurrence overlay diagram based on 3WD in Fig. 9 and the co-occurrence overlay diagram based on 3WD in generalized IF environments in Fig. 10 can be obtained.

Figure 9 demonstrates the significance of model, attribute reduction, and rough sets in the domain of 3WD research.

Figure 10 illustrates the significance of model, three-way decisions, and three-way decision in the research of 3WD in generalized IF environments.

#### Citations

**Fig. 11:**
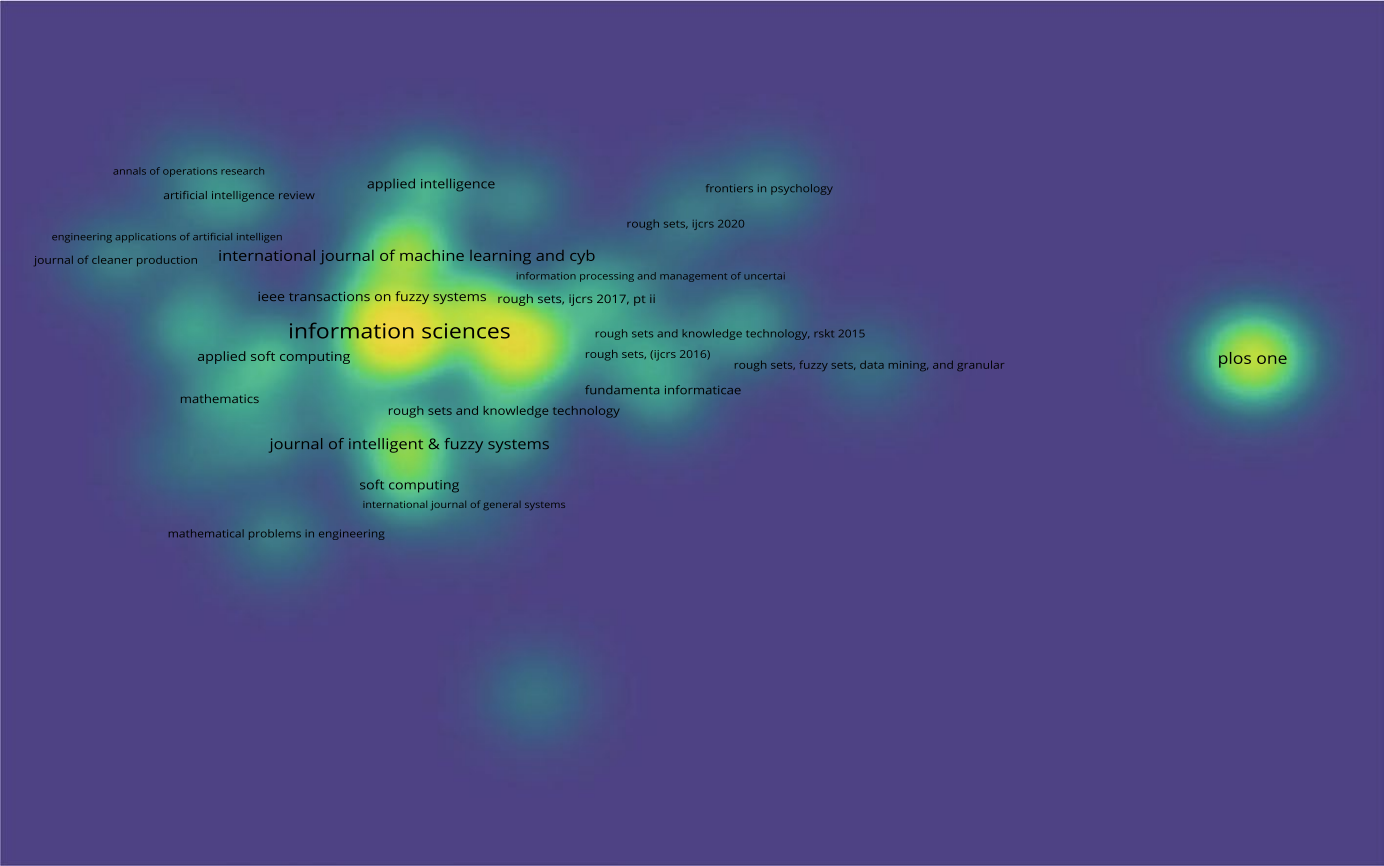
Citation density for 3WD

**Fig. 12:**
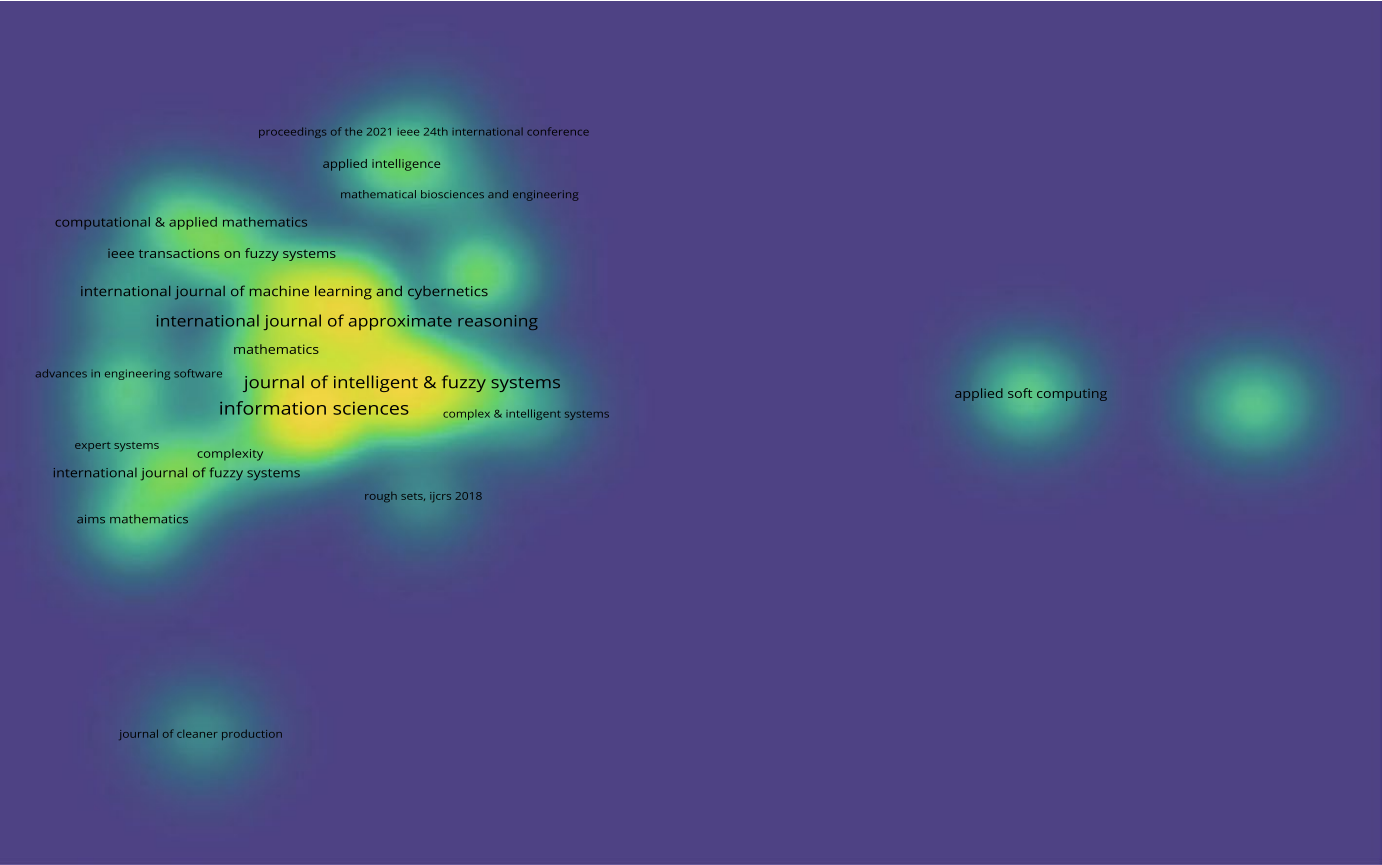
Citation density for 3WD in generalized IF environments

Next, the citation density diagram based on 3WD in Fig. 11 and the citation density diagram founded on 3WD in generalized IF environments in Fig. 12 can be obtained.

Figures 11 and 12 indicate that the journal Information Sciences has the highest number of publications.

### Reference analysis

**Fig. 13:**
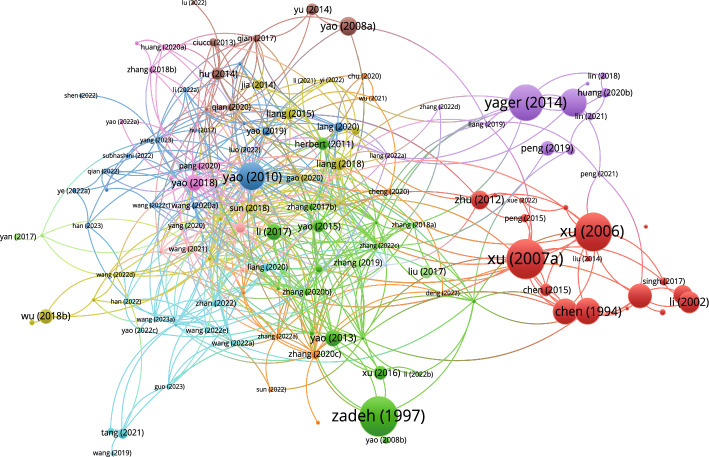
Citation document networks of references

**Fig. 14:**
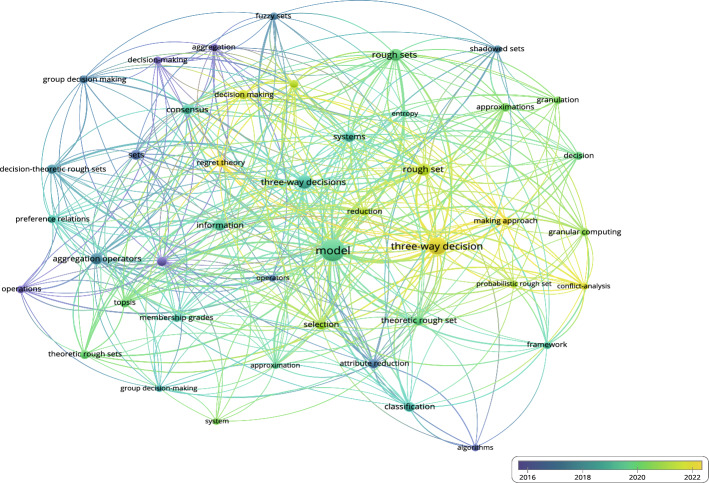
Co-occurrence overlay of references

**Fig. 15:**
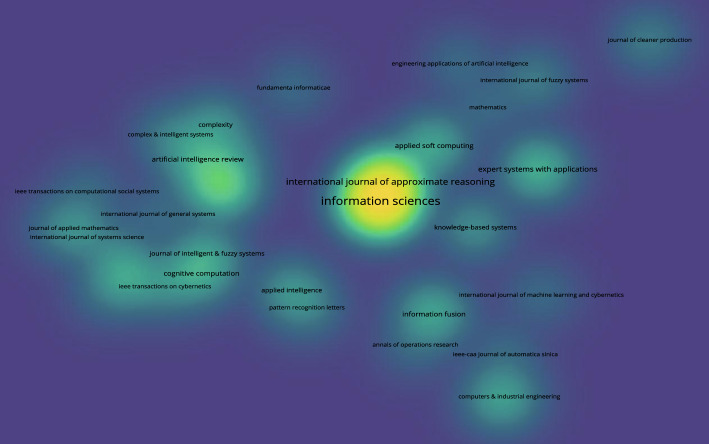
Citation sources density of references

This paper conducts a statistical analysis of 168 references, while visual analysis was performed on 154 references (excluding (Buxton et al. 2008; Chen et al. 2021; Zadeh et al. 1979; Hobbs 1985; Lin 1997; Atanassov 1986; Zadeh 1965; Atanassov and Gargov 1989; Pawlak 1982; Bai et al. 2020; Ghaderi et al. 2020; Mousavi et al. 2015; Gitinavard and Akbarpour Shirazi 2018; Li et al. 2023b)). Figure 13 shows the citation documents network of references, Fig. 14 shows the co-occurrence overlay of references, and Fig. 15 shows the citation sources density.

From the citation documents network graph, it is evident that the papers by Xu, Zadeh, Yao, Yager have played a crucial role in their research field and had a significant impact on subsequent research.

Figure 14 illustrates the significance of model, three-way decisions, and three-way decision in the research of references.

Figure 15 indicates that the journal titled Information Sciences owns the highest number of publications.

## Research progress

In the following, this paper provides a brief analysis of ten recent 3WD papers conducted in generalized IF environments over the past three years. These papers have broadened the research scope by exploring various aspects, including loss functions, conditional probabilities, and thresholding. Following that, this paper delves into the distinctions between these methods, along with their unique characteristics and inherent limitations.

### Decision-making models

Jiang and Hu (2021) employed the evaluation values of attributes to determine the relative cost and revenue functions of the alternatives. Then, considering the problem involving multiple attributes in MAGDM, the aggregated cost and revenue functions were calculated and integrated to get the relative profit function of the alternative. On this basis, the overall profit function was obtained through integration. Furthermore, the conditional probability was obtained by using the IF TOPSIS method. In conclusion, a three-way investment decision model was proposed and implemented to evaluate the coalfield investment case. The model’s requirement time is *O*(*mnf*). However, the weight of the model is a subjectively given factor, and different weights may affect the final ranking result, thus calculating the weights objectively is highly significant.

Liu et al. (2022) introduced LIF numbers into loss functions, discussed a new ranking method, and built an LIF DTRS model based on the ranking method. Then, a single optimization model was created to count the threshold value, and Karush-Kuhn-Tucker was utilized for proving the distinctiveness of the optimum solution of the proposed approach. Ultimately, the effectiveness of the advocated approach was indicated by providing a case study of human–machine task allocation. The model’s requirement time is *O*(*mn*). However, the model can only assign alternatives to different regions, and cannot rank alternatives. Sorting the alternatives is necessary in the actual decision-making issues to minimize losses in the process of making a decision.

Jia and Liu (2021) introduced a novel approach to construct a relative loss function by utilizing the idea of an ideal solution. They proposed two ideal solutions that focus on attributes. The first ideal solution approach involves assigning a separate loss function to each attribute. In this approach, each attribute is assigned its own loss function, and by summing up the losses across all attributes, the final loss function can be derived. The second approach to construct a relative loss function, which involves a global ideal solution perspective. Measuring the distance between the alternative solution and the global ideal solution is how the final loss function is determined in this framework. The first ideal solution method is employed in fuzzy environments with varying risk avoidance coefficients. The second ideal solution approach is employed in an IVIF environment with a constant risk avoidance coefficient. Model’s requirement time is *O*(*mn*). However, it should be noted that the global ideal solution approach has a limitation, as it does not take into account the losses between the ideal solution and each attribute. The introduction of bias during the process is inevitable when attribute weights are subjectively set, given that each attribute’s influence on the decision result is determined by its weight.

Xue et al. (2022) studied four kinds of IHF multigranulation rough sets, namely pessimistic, optimistic, optimistic-pessimistic and pessimistic-optimistic types, proposed a new IHF cross-entropy, and employed the TOPSIS method to compute the conditional probabilities for all four cases. The authors employed the subtraction function to obtain the threshold and subsequently derived the corresponding positive, negative, and boundary domains. This process resulted in the creation of four types of 3WD models. The model’s (take the three-way rule algorithm built upon an optimistic multigranulation rough IHFS as an illustration) requirement time is *O*(*mnf*). However, this method only gives the classification results and does not select the optimal results.

Wang et al. (2022a) first determined the probability dominance class according to the given confidence level, then obtained the objective fuzzy state set from the initial data and then computed the conditional probabilities using the probability dominance relation and the fuzzy state set. Further, to reduce decision risk, six relative loss functions were considered for each alternative. In conclusion, the authors offered a three-way IF MAGDM framework founded upon probabilistic dominance relationships and applied it to the case of parts supplier selections. The model’s requirement time is $O(m{n^2})$.

Song et al. (2022) studied a hybrid MAGDM method. First, attribute values of different forms were converted into the IVIF number form with minimum information distortion, and then expert weights were computed by using IVIF entropy and cross-entropy to create a group comprehensive evaluation matrix. Second, the IVIF entropy was utilized to count the weights of attributes. Moreover, the improved VIKOR means founded on grey correlation analysis was used to count the conditional probability, and the threshold was determined through an optimization method. In the end, a hybrid multi-attribute 3WD approach was constructed founded on the enhanced VIKOR method. The model’s requirement time is *O*(*mnf*). However, this approach does not do sorting, which can help decision-makers choose some suitable alternatives.

Yi et al. (2022) first used PF similarities to calculate conditional probabilities. Then the concept of action utility functions was introduced, and a risk measurement matrix was constructed founded on an S-shaped utility functions. Further, with utility maximization as the goal, the optimization function was introduced to calculate the threshold value. Finally, the PF 3WD approach with S-shaped utility function was constructed. The model’s requirement time is *O*(*mn*). However, the amount of experts in this paper is single, which is inconsistent with the existence of multiple experts in real life.

Liang et al. (2022b) studied the integration scheme for the interval-valued q-rung orthopair fuzzy set (IVq-ROFS), and at the same time modified the integration region to remove meaningless points in advance. Then an equivalence class was constructed based on the similar class to solve the conditional probability. Further, the IVq-ROF integral function was used to aggregate the loss function. Finally, three classification rules were obtained by comparing the expected loss function. The model’s requirement time is $O(m{n^2})$. However, this method only provides classification results for alternatives, and does not identify the optimal solution among them.

Wang et al. (2022c) developed a 3WD methodology founded upon RT in HF environments. The model combined RT with HFSs to calculate perceived utility value, and then improved the regret-rejoice function based on Taylor expansion. Finally, it is classified built upon the PROMETHEE I method. The model’s requirement time is $O(4m{n^2})$.

Dai et al. (2023) captured the experts’ preferences for each criterion from two perspectives of membership degrees and non-membership degrees, and then discussed a novel conditional probability that relied on IF similarities and constructed a loss function calculation approach founded upon the IF concept. Finally, the minimum risk rule was established. The model’s requirement time is *O*(*mn*). This approach is limited to decision-making problems that have IF information, and cannot be used in other types of problems. This implies that experts need to convert the actual data into data with IF information before utilizing this approach to make decisions.

### Discussions

Next, this paper analyzes the above decision-making methods.

Table 4 shows the differences between the above methods in contexts, loss function extensions, conditional probability extensions, ranking and classification.

**Table 4:** The differences between different approaches

Different approaches	Backgrounds	Loss function extensions	Conditional probabilities	Group decisions	Ranking
Jiang and Hu (2021)	IF information system	$\times$	$\checkmark$	$\checkmark$	$\checkmark$
Liu et al. (2022)	LIF information system	$\times$	$\times$	$\times$	$\times$
Jia and Liu (2021)	IVIF information system	$\times$	$\times$	$\times$	$\times$
Xue et al. (2022)	IHF information system	$\times$	$\checkmark$	$\checkmark$	$\times$
Wang et al. (2022a)	IF information system	$\times$	$\checkmark$	$\times$	$\checkmark$
Song et al. (2022)	Hybrid multi-attribute information system	$\times$	$\checkmark$	$\checkmark$	$\times$
Yi et al. (2022)	PF information system	$\checkmark$	$\checkmark$	$\times$	$\checkmark$
Liang et al. (2022b)	IVq-ROF information system	$\times$	$\checkmark$	$\times$	$\times$
Wang et al. (2022c)	HF information system	$\times$	$\times$	$\times$	$\checkmark$
Dai et al. (2023)	IF information system	$\checkmark$	$\checkmark$	$\times$	$\checkmark$

As is illustrated in Table 4, the following conclusions can be obtained:

1. The 3WD model is applied to different IF environments, such as IHF environments (Xue et al. 2022), IVIF environments (Jia and Liu 2021), and LIF environments (Liu et al. 2022).
2. Part of the 3WD model only pays attention to classification results whereas ignoring ranking results, such as the Xue et al.’s (Xue et al. 2022) method, (Liu et al. 2022) method and Liang et al.’s method (Liang et al. 2022b).

To provide a more clear and intuitive comparison of the aforementioned methods, this paper presents their characteristics and limitations in Table 5.

**Table 5:** Characteristics and limitations of different approaches

Different approaches	Characteristics	Limitations
Jiang and Hu (2021)	* A relative profit function is constructed by combining the relative cost with benefit functions. This function is then integrated to obtain the overall profit function. * Use the TOPSIS method to acquire conditional probabilities. * Relevant profit values and preference rules are utilized to sort alternatives, assisting experts in selecting suitable options based on the specific circumstances	* The subjective assignment of attribute weights can result in one-sided decision outcomes that do not align with actual situations
Liu et al. (2022)	* Better performance based on less computation and less time to solve the threshold. * Two nonlinear optimization models are designed to determine the risk loss threshold pairs. * It can be extended to other forms of DTRSs	* This method assumes that the conditional probability falls between 0 and 1, without taking into account the actual conditional probability of the object. * The loss function of this method was given subjectively and was not tested with UCI data sets
Jia and Liu (2021)	* Three ideal solutions are studied. * The IVIF relative loss function is established through the ideal solution. * Two types of IVIF 3WD models are proposed built upon the individual ideal solution and global ideal solution perspectives	* The loss function based on the global ideal solution does not take into account the loss between the ideal solution and each attribute
Xue et al. (2022)	* A new IHF cross entropy is defined. * Use the TOPSIS method to compute conditional probability. * The decision threshold is calculated according to the subtraction function	* This approach does not consider the boundary domain of decision case alternatives, and it overlooks the weight of each granularity
Wang et al. (2022a)	* The conditional probability can be determined by utilizing the probabilistic dominance relation. * Objective calculation of fuzzy state set reduces the influence of subjective factors. * By transforming the overly strict equivalence relation into a dominance relation, and building the dominance relation founded upon this transformation, the model has a greater impact on sorting results. * The relative loss function is calculated in light of the evaluation value, which reduces the decision risk of subjective loss functions	* By converting IF numbers into fuzzy numbers and obtaining the relative loss function, this method focuses on processing fuzzy numbers in the model
Song et al. (2022)	* The hybrid multi-attribute environment is more compatible with real-life situations and has greater versatility. * Determining expert weights from the perspectives of IVIF entropy and cross-entropy is a more reasonable and objective approach. * The utilization of the improved VIKOR method, which incorporates grey correlation analysis, to calculate conditional probability enhances the scientific rigor of the calculation process	* The determination of expert and attribute weights often only considers a single form of IVIF entropies. * Only categories are discussed, but sorting is not explored
Yi et al. (2022)	* From the perspective of cognition, a new scoring function is defined. * Calculate conditional probabilities based on similarity and proximity. * The risk measurement function is created, which effectively reduces the subjective error, and the model has low delay cost. * The threshold is calculated using the optimization model	* The number of experts in this method is single, while in real life, more than one expert is often needed. * The attribute weight of this method is subjectively given
Liang et al. (2022b)	* A model for converting similar classes into equivalence classes is constructed, which provides an idea for solving equivalence classes in other fuzzy environments where distance is not transitive. * Set up correction function to remove nonsense points in advance. * Taylor series is used to replace complex integral functions	* The model only discusses the classification results, and does not explore the sorting results. The sorting results can help experts select appropriate alternatives
Wang et al. (2022c)	* Founded on Taylor’s expansion, the regret-rejoice function is improved. It satisfies experts’ sensitivity to regret and rejoice while preserving real-world uncertainty. * To alleviate subjectivity in decision-making, a classification method founded upon PROMETHEE I is offered	* The model is developed within an HF environment and cannot directly address issues in diverse uncertain contexts. * This model does not consider the influence of risk avoidance behavior in the face of income and loss on the final decision result
Dai et al. (2023)	* The IF loss function, which takes into account both membership degree and ND, provides a more accurate representation of real-world loss problems. * The conditional probability calculation method based on IF similarity meets the minimum requirement of experts for alternatives on the one hand, and measures the quality of alternatives well on the other hand	* This method can only be used in decision-making scenarios where IF information is available, requiring the decision-maker to have access to data represented in the form of IF numbers. This may require experts to convert the actual data they have into data with IF information before making a decision

## Challenges and future research directions

Next, this paper will introduce several future research directions.

### The determination of weights

Attribute weighting is a critical area of study in MAGDM problems. The concept of weights pertains to the vital of each indicator during the decision-making phase. This relative measure signifies the relative contributions of the indicators to the evaluation process. The weight of different attributes represents their relative importance during the decision-making phase, and varying weights can significantly impact the final decision result. The subjective weighting approach is determined by experts based on subjective experience, such as analytic hierarchy process (Mathew et al. 2020). Subjective weighting method has strong subjectivity, whereas objective weighting methods are based on mathematical algorithms that derive weights directly from the original data without relying on subjective judgments. The entropy weight method (Wang et al. 2022c) is one of these objective methods. Objective weighting method does not depend on subjective judgment and has strong theoretical basis of data. However, when assigning attribute weight, To make accurate evaluations, it is imperative to consider both the attributes’ internal connections and the experts’ level of authority (Song et al. 2022). Therefore, in order to obtain reasonable attribute weights, some researchers combine subjective empowerment methods and objective weighting methods to determine attribute weights, and then make up for the shortcomings caused by single empowerment. The above combinatorial weighting method is a relatively reasonable method for solving attribute weights, but how to determine the weights between the two weighting methods needs to be further explored.

In MAGDM, not only attribute weights but also expert weights are involved. Different attributes hold varying levels of importance for specialists, and different specialists may have varying opinions regarding the significance of the same attribute. Therefore, the determination of attribute weights and expert weights is critical. How to determine the weight of different experts under different attributes is an important issue.

### The attribute reduction founded on 3WD in incomplete generalized IF environments

The rough set theory is mathematical framework utilized for addressing imprecise, inconsistent, and incomplete information (Pawlak 1982). Within the realm of the rough set theory, attribute reduction (Cheng et al. 2020) is considered a fundamental area of study. The fundamental concept of attribute reduction (Qian et al. 2017) is to eliminate redundant attributes from data and obtain a reduced table that contains only relevant attributes. This condensed table does not compromise the classification efficiency or decision-making capability of decision-making information; rather, its objective is to enhance the classification performance. Existing 3WD attribute reduction methods have made significant progress in improving the capability of decision frameworks by reducing the amount of attributes in the data set. However, there is still potential for improvement in the following areas:

1. Most existing attribute reduction (Yao and Zhao 2008) algorithms are based on static information systems, where the needs of the system and users are assumed to be fixed and unchanged. Nevertheless, in realistic decision-making scenarios, the needs of the information system and users may change over time due to various factors such as evolving preferences, changing requirements, and new information becoming available. Therefore, it is necessary to study dynamic 3WD attribute reduction.
2. The existing experimental data set of attribute reduction is relatively complete, but incomplete data are common in practical problems (Zhan et al. 2022; Deng et al. 2022). Consequently, it is very sense to discuss the dynamic 3WD attribute reduction under incomplete generalized IF environment (Hussain et al. 2021, 2022b).
3. Current methods do not classify boundary domain data further, therefore, new techniques could be developed to handle boundary domain data and improve the classification accuracy of 3WD methods.

### Research on consensus decision-making in incomplete generalized IF systems

In reality, people are faced with various decision-making problems on a regular basis. However, due to differences in cultural level and social background, different experts may have different perspectives and ideas on the same issue. This can pose a challenge in reaching a unanimous decision. The group consensus decision model is a useful tool that can help coordinate conflicting viewpoints and ideas from different experts when making decisions. This can help to facilitate agreement and ultimately lead to a consensus decision (Wu et al. 2019; Zhu et al. 2023b). Hence, researching the consensus decision model for three-way groups under an incomplete generalized IF environment (Lin et al. 2021) is imperative.

Consensus reaching is a process where experts come to an agreement on decision results founded on their individual preferences. The process of reaching consensus generally includes two parts: 1) consensus measure (Yan et al. 2017); 2) feedback adjustment (Wu et al. 2018b). Therefore, the research direction faces the following problems.

1. A consensus measure is a way of quantifying the level of agreement or similarity between individual opinions and group opinions. It is used to evaluate the degree to which a group has reached a consensus decision. Existing consensus measurement mainly includes two categories: hard consensus degree and soft consensus degree. Hard consensus requires complete agreement, however complete agreement is difficult in practical problems, thus the soft consensus is more reasonable. However, it is worth studying how to reasonably solve the group consensus level in incomplete generalized IF environments.
2. In the feedback process, it is important to preserve the initial preferences of experts as much as possible, thus how to create a feedback mechanism with minimal adjustment costs is a difficult challenge.
3. The loss function is a key element of the 3WD. To ensure that the loss function accurately reflects the opinions and preferences of all group members, it is essential to create a loss function matrix through group consensus.

### The analysis of large-scale group decision-making in generalized IF systems

Over recent years,the rapid pace of development in information technology and the escalating complexity of decision-making issues has brought about the emergence of new characteristics among participants in group decision-making. These features include group scale, information diversification, and complicated behavior, among others. As a result, researchers have begun to focus on the development of large-scale group decision-making as a specific research direction (Tang and Liao 2019). large-scale group decision-making has the following characteristics: 1) More decision-making experts come from all walks of life and have different knowledge and experience. 2) Experts can allow decisions to be made in different places at different times. 3) There may be interpersonal dynamics among decision-making experts, making it challenging to achieve a high level of consensus (Wang et al. 2019). There are several challenges with the study:

1. Reduce the dimension. Dimension reduction is a crucial aspect of large-scale group decision-making as it assists in reducing the cost and complexity of the decision-making process. Clustering analysis is widely regarded as the most commonly utilized approach in dimension reduction analysis. Clustering analysis (Wu et al. 2018a) is the process of grouping individuals with similar preferences into an organization.
2. Consensus. The process of reaching consensus involves narrowing the gap between experts’ opinions (Zhang et al. 2020a). However, in real world, some experts may not be willing to revise their initial opinions, thus non-cooperative behaviors need to be considered. Second, the decision-making problem with complex preference information may need to be considered in practical application, thus it is worth studying how to reach consensus in this case.

### Sequential 3WD in generalized IF systems

At present, based on the two dimensions of time and space, 3WD can be divided into static and dynamic two kinds, and most of the above discussion is static 3WD, so this paper needs to consider how to do further research on dynamic 3WD. Sequential 3WD is the representative of dynamic 3WD. Sequential 3WD (Yang et al. 2023; Han and Zhan 2023) is a multi-level grain size model, from coarse to fine grain. The existing decision model does not consider the sequential 3WD under the environment of IF model. Therefore, it is meaningful to study the sequential 3WD under the generalized IF environment (Bai et al. 2020; Jin et al. 2023). This paper briefly describes the challenges that this model will encounter.

1. How to integrate IF numbers of different stages effectively. The combination of IF numbers greatly influences the ultimate effectiveness of the decision-making model. How to find a stable fusion of IF numbers is challenging.
2. Currently, the ordered 3WD models primarily obtain the threshold value from two angles, the primary aim is to enhance the precision of the model in classification, while the secondary objective is to decrease the level of uncertainty related to the boundary field during the classification process. In real life, experts aim for a model that not only achieves high classification accuracy but also minimizes uncertainty in the boundary domain. However, in the sequential 3WD, the above two perspectives are difficult to reach the optimal at the same time, so how to balance the contradiction between the two, and then select the decision threshold is worth studying.
3. Existing cost-sensitive sequential 3WD improves the effectiveness of 3WD from the perspective of grain computation. However, this method still has some problems in the selection of optimal grain size, which needs further improvement.

### The combination of 3WD and machine learning

Machine learning is a discipline that aims to optimize the performance of computer systems by leveraging the power of data and experience. It involves constructing and training models to enable computers to learn and adapt automatically without explicit programming instructions. Machine learning leverages techniques and methods from various fields, including statistics, data mining, and AI, to analyze and interpret data, uncover patterns and trends, and facilitate accurate predictions and informed decision-making. The advancement of machine learning has propelled the progress of AI and has made a notable contribution in practical applications.

#### Convolutional neural networks

Convolutional neural networks are a class of feedforward neural networks characterized by local connections and weight sharing. They are widely recognized as a prominent algorithm in the field of deep learning. Convolutional neural networks can handle inputs of different sizes, and have strong feature extraction capabilities, requiring fewer parameters and lower complexity, making them a good classification model (Yu et al. 2022). However, convolutional neural networks cannot handle uncertainty in data. 3WD, as a theoretical model that conforms to human cognition, can handle uncertainty problems well. Therefore, combining convolutional neural networks with 3WD is meaningful, but the model faces the following challenges:

1. Determining the threshold. Determining the threshold is a key step in 3WD (Wang et al. 2023b). Different thresholds will have an impact on classification results. It is essential to select an optimal threshold, limit the number of iterations to a reasonable range, and ensure that the classification results have a relatively high level of accuracy.
2. After using 3WD for region partitioning, delayed decisions are needed for data with insufficient information. How to use convolutional neural networks to further classify data in boundary domains, so that the classification results have only two accept decisions and reject decisions is important (Subhashini et al. 2022).

#### Three-way classification

Feature selection (Li et al. 2023a) is a commonly used technique in machine learning that selects meaningful features from raw data to improve model accuracy and generalization. The Naive Bayesian algorithm is a simple and prevalent classification method, widely and effectively applied in the field of supervised learning (Yao and Zhao 2008). The Naive Bayesian algorithm requires the assumption that the values of the attributes describing the data are mutually independent under a given category, meaning that the value of any attribute does not depend on other attributes. The 3WD approach is a highly effective tool for managing uncertain information. Combining feature selection, the Naive Bayes algorithm and 3WD can better address complex issues such as uncertain probability distributions and correlations between features, thereby obtaining more accurate results in classification tasks (Wu et al. 2021). This combination optimizes model performance, reduces computational complexity, and improves classification efficiency. However, this research direction faces some challenges.

1. Traditional feature selection methods (Sangaiah et al. 2022) typically assume a linear relationship between features and the target variable. However, real-world data often exhibits complex nonlinear relationships, necessitating feature selection (Li et al. 2023b) methods that can discover and leverage these nonlinear relationships to enhance model performance.
2. Conditional probability is a crucial component in 3WD, and calculating it accurately is a significant challenge in this research field, as it is essential for improving classification accuracy.
3. The Naive Bayesian algorithm is a non-incremental algorithm, but it can be limited in analyzing massive data as the data size increases. Therefore, combining incremental algorithms with the Naive Bayesian algorithm is necessary to avoid repeated training and probability recalculations, and to boost the scalability of the algorithm.
4. The assumptions underlying the Naive Bayesian algorithm may not always be valid in real-world scenarios. To address this issue, additional features can be introduced, and techniques such as correcting distribution bias can be employed to minimize the cases where the assumptions are not valid and improve classification performance.

#### Three-way clustering

Clustering analysis is a type of unsupervised learning in machine learning (Sangaiah et al. 2023). Clustering involves partitioning a data set into distinct groups or clusters founded on specific criteria, such that objects within the same cluster exhibit higher similarity to one another, whereas objects assigned to different clusters exhibit a lower degree of similarity. Clustering results are commonly presented as a unified set, wherein each object is allocated to a specific cluster based on its similarity with other objects in that same cluster. It is noteworthy that the original statement that clustering results are represented as a single set is generally true, but there may be cases where an object’s membership to a cluster is ambiguous. In such cases, it may be more appropriate to use a three-domain representation to create three-way clustering (Yu et al. 2014; Zhang et al. 2023b), allowing for objects that exhibit mixed characteristics from multiple clusters or are only loosely associated with a particular cluster. In accordance with the context and characteristics of the data, a three-way clustering (Chu et al. 2020) approach may offer a more nuanced portrayal of the inherent framework of the data, compared to the traditional two-way clustering approach. However, using three-way clustering (Guo et al. 2023) may pose certain challenges:

1. Determining the optimal amount of clusters is a critical aspect of clustering research. For some clustering algorithms, such as K-means, the amount of clusters must be specified in advance. If the amount of clusters is set incorrectly, the resulting clustering may be ineffective. Therefore, it is vital to identify the optimal amount of clusters that accurately reflects the inherent patterns and variability in the data. Several techniques can be used to determine the optimal amount of clusters, such as the elbow method, silhouette analysis, and gap statistic.
2. Three-way clustering (Sun et al. 2022) relies on 3WD, requiring appropriate thresholds to partition experimental data into core, edge, and external regions. However, most thresholding methods for the three clustering algorithms are subjectively determined and one-sided. Obtaining thresholds objectively is a worthwhile research direction.
3. While there has been extensive research on static data sets, there is a need for further investigation on dynamic data sets.

#### Game-theoretic rough sets

Game theory (Zhang and Yao 2020) is a mathematical framework that offers a formal approach to analyzing strategic interactions and decision-making in situations where multiple agents have competing interests. Rather than seeking to find a compromise solution among contradictory choices, game theory provides a means of understanding how rational agents make decisions and interact with each other. Game-theoretic rough sets (Yao and Azam 2015), which involve establishing various games, can help determine a reasonable threshold for decision-making. The key to game-theoretic rough sets is the trade-off between the deterministic domain (positive and negative domains) and the uncertain domain (boundary domain). After a series of iterations, a threshold pair that satisfies Nash equilibrium is ultimately obtained in game-theoretic rough set. In a Nash equilibrium, each player in a game chooses a strategy that is best for them given the strategies chosen by the other players. In other words, no player can unilaterally change their strategy and improve their outcome. Nash equilibrium is a crucial concept in game theory, as it provides a stable state where the choices of all players are mutually consistent. The process of determining the optimal threshold in game-theoretic rough set is determined by balancing accuracy with positive, negative, and boundary domain size. However, combining game theory rough sets with 3WD (Zhu et al. 2023a) presents several challenges. These challenges include:

1. The selection structure of decision conditions. Nash equilibrium (Herbert and Yao 2011) can be employed to ascertain the conditions that establish an equilibrium among various game outcomes.
2. High computational complexity. Due to the need to consider multiple factors, this approach has a high computational complexity, requiring significant time and computing resources.
3. The outcomes predicted by existing game-theoretic rough set models are often founded on idealized assumptions that may not hold up in the real world. As a result, decision-makers facing complex decision-making problems may need to continually simulate and refine their strategies to achieve better outcomes. This requirement has led to the development of game theory models that take into account the bounded rationality.

### The combination of 3WD and AI

AI is a scientific and technological field dedicated to simulating human intelligent behaviors. It encompasses the development of intelligent systems capable of perceiving, understanding, learning, reasoning, and making decisions to successfully complete diverse tasks. Intelligent decision-making is a crucial area within the field of AI. By harnessing AI technologies for intelligent decision-making, it becomes possible to mitigate subjective biases. Moreover, this enables the enhancement of decision precision and coherence to a higher level. Furthermore, Superior solutions can be unearthed in intricate decision-making scenarios Therefore, AI’ application has broad applicability and importance, which can improve people’s lives and work, and promote social development and progress.

#### Digital twin

Digital twin (Tao et al. 2022) is a virtual model that accurately replicates a physical entity. This technology enables the continuous collection and intelligent analysis of operational data, providing a more comprehensive understanding of the entity’s behavior. By combining digital twin with the 3WD approach, this paper can effectively handle information uncertainty and enhance decision-making accuracy and efficiency. For instance, in the field of urban management, digital twin is a prime example of how this technology can be used. Real-time monitoring of traffic flow can be achieved with digital twin, while data analysis and simulation techniques from the 3WD approach can be used to propose solutions for improving traffic congestion and achieving smart city management. In conclusion, the fusion of digital twin and the 3WD approach (Yao et al. 2022a) can enable more intelligent and precise decision-making, ultimately leading to better solutions in various fields.

#### Metaverse

Metaverse (Deveci et al. 2023) is a virtual reality space that combines the digital world with the real world on a virtual platform. Through metaverse, users can engage in various activities and interactions in a digital virtual space, including gaming, socializing, commerce, and more. On the other hand, the 3WD approach is a data-driven and machine learning-based decision-making method that enables more accurate and efficient decision-making through continuous learning and optimization. By integrating 3WD with metaverse, this paper can further enhance the accuracy and efficiency of decision-making, bringing greater value to the various activities and interactions within metaverse. Specifically, 3WD can leverage the data within metaverse for analysis and processing, enabling more accurate and comprehensive decision support. Furthermore, 3WD can continuously learn and optimize, further enhancing the precision and efficiency of decision-making. For instance, in the gaming domain, metaverse can provide a wealth of game data and player behavioral data. 3WD can leverage this data for analysis and processing, thus offering more accurate game experiences and optimization suggestions for game processes. To summarize, the integration of 3WD means with metaverse can enable more precise management and intelligent decision-making, providing greater value and experiences for various activities and interactions within metaverse.

#### AI generated content

AI generated content (Wu et al. 2020) refers to the technology that utilizes AI to create various forms of content, such as paintings, text, and more. The 3WD method (Yao et al. 2022b) is an approach that divides the overall problem into three parts, and then makes decisions accordingly. By integrating AI generated content with 3WD process, this paper can better leverage AI technology while ensuring the quality and accuracy of the generated content. First, this paper can utilize AI-generated content to improve production efficiency and reduce workload. However, this paper must also consider the fairness and accuracy of the generated content. Therefore, it is crucial to incorporate the concept of 3WD in the content generation process to ensure that the content meets the criteria of interest, fairness, and efficiency. In summary, integrating AI generated content with the concept of 3WD can assist us in leveraging AI technology effectively while ensuring the quality and accuracy of the generated content, thereby better meeting user needs and requirements.

### The application of 3WD

In today’s rapidly-evolving society, the decision-making process has become more intricate and challenging, particularly when dealing with highly complex and multifaceted issues. As a result, the 3WD method has gained significant traction and is increasingly being recognized as a valuable decision-making tool. With the aid of the 3WD approach, decision-makers can make more sound and rational judgments, even when confronted with incomplete or inadequate information. This, in turn, leads to higher quality outcomes and reduces the likelihood of mistakes, as the improvement during decision-making processes is realized. Hence, the 3WD approach is being widely adopted in numerous domains, including talent acquisition (Ye et al. 2021), disease diagnosis (Wang et al. 2023a), and forest fire management (Wang et al. 2022b).

As information technology and AI continue to advance, the 3WD method (Ding et al. 2023) will find even broader and richer applications. For instance, the finance industry can leverage this method to evaluate market trading trends, providing investors with the ability to make more precise investment decisions. Similarly, in the development of corporate strategies, the 3WD method can assist managers in generating preliminary strategic options, which can then be compared based on various factors such as market environment, competitors, and other relevant factors to identify the optimal strategy.

In the field of management decision-making, the 3WD method can be applied in various contexts. Specifically, in supply chain management decisions, businesses can utilize this method for multi-objective optimization in supplier selection (Hajiaghaei-Keshteli and Fathollahi Fard 2019; Hajiaghaei-Keshteli et al. 2023b), logistics strategy formulation, and inventory management. (Hajiaghaei-Keshteli and Fathollahi Fard 2018) have made significant progress by employing a two-stage stochastic bi-level programming model to study distribution network problems. Building upon this foundation, the organic integration of the 3WD method with this model can lead to a comprehensive decision support approach. This approach not only addresses multiple objectives and constraints within this problem but also extends its applicability to a broader spectrum of management decision-making domains, achieving holistic optimization of the decision-making process. In project investment decisions, businesses can employ the 3WD method to evaluate expected returns, risks, and opportunity costs for different projects, thereby achieving the optimal allocation of investments in a portfolio. By combining the 3WD method with the specific problem modeling, its quantitative advantages in management decision analysis can be leveraged to assist decision-makers in making well-rounded decisions that take into account various factors in complex environments.

To conclude, the 3WD method is expected to exert a significant influence on diverse domains in the future. It will enable individuals to make better-informed decisions when confronted with incomplete information, leading to enhanced decision-making quality and reduced chances of errors. As information technology and AI continue to evolve, the 3WD method will undoubtedly find even broader and more diverse applications, taking on an increasingly critical component in decision-making processes.

## Conclusions

This study endeavors to investigate the 3WD model based upon the generalized IF environment (Lin et al. 2018; Huang et al. 2020a) and analyze its value and significance in both theoretical research and practical applications. The paper highlights the characteristics and limitations of current research while emphasizing the necessity for further exploration in combining the 3WD theory with generalized IFSs in future studies. It also underscores the importance of expanding the application of this combination in various fields. To summarize, the present investigation provides a valuable foundation and basis for prospective scholarly inquiries.

This paper provides a systematic review, analysis, and summary of recent advancements and developments in the field of 3WD and generalized IFSs. Initially, a review was conducted on the fundamental theoretical foundations of 3WD, followed by a comprehensive discussion of its achievements in theoretical framework, methods, and practical applications. Subsequently, the basic theoretical foundations of IFSs were reviewed, and its achievements in theoretical framework, methods, and practical applications were comprehensively discussed. Then, a review was presented on relevant papers regarding 3WD in generalized IF environments. Afterwards, the bibliometric analysis of the literature pertaining to these theories and the references of this paper was conducted using bibliometric methods. Additionally, an in-depth examination of ten papers from the past three years is presented. In conclusion, the paper puts forward seven potential research topics for future investigation. Exploring these topics could enhance our knowledge of these technologies and their potential applications.

This study has not yet integrated the 3WD model with transfer learning and supervised learning in machine learning. There is immense potential for future research to explore combining the 3WD model with these machine learning approaches to achieve more comprehensive and efficient problem solving. Additionally, integrating the 3WD model with emerging technologies such as the metaverse and latest models like GPT warrants further investigation. These explorations could uncover new possibilities for solving complex problems and improving predictive performance, holding academic and practical significance for related fields.

## Footnotes

1 http://webofscience.com/
